# RAB2A promotes cervical cancer progression as revealed by comprehensive analysis of HPV integration and proteome in longitudinal cervical samples

**DOI:** 10.1002/ctm2.767

**Published:** 2022-03-28

**Authors:** Yifan Meng, Shitong Lin, Yi Zhou, Xiao Yi, Ping Wu, Qing Zhang, Weigang Ge, Canhui Cao, Peipei Gao, Wenhua Zhi, Ting Peng, Juncheng Wei, Wencheng Ding, Ding Ma, Guoliang Li, Qin Yang, Tiannan Guo, Xi Zeng, Peng Wu

**Affiliations:** ^1^ Department of Gynecologic Oncology State Key Laboratory of Oncology in South China Collaborative Innovation Center for Cancer Medicine Sun Yat‐sen University Cancer Center Guangzhou Guangdong China; ^2^ Department of Gynecologic Oncology Tongji Hospital, Tongji Medical College Huazhong University of Science and Technology Wuhan Hubei China; ^3^ Cancer Biology Research Center (Key Laboratory of the Ministry of Education) Tongji Hospital, Tongji Medical College Huazhong University of Science and Technology Wuhan Hubei China; ^4^ Agricultural Bioinformatics Key Laboratory of Hubei Province and Hubei Engineering Technology Research Center of Agricultural Big Data College of Informatics Huazhong Agricultural University Wuhan Hubei China; ^5^ Westlake Omics (Hangzhou) Biotechnology Co. Ltd. Hangzhou Zhejiang China; ^6^ Department of Obstetrics and Gynecology Qilu Hospital of Shandong University Jinan Shandong China; ^7^ Department of Pathology Tongji Hospital, Tongji Medical College Huazhong University of Science and Technology Wuhan Hubei China; ^8^ Key Laboratory of Structural Biology of Zhejiang Province School of Life Sciences Westlake University Hangzhou China; ^9^ Institute of Basic Medical Sciences Westlake Institute for Advanced Study Hangzhou Zhejiang China

Dear editor,

To date, no relevant research has explored the dynamic changes in omics to investigate the mechanisms driving the process of cervical cancer malignant transformation. We explored the role of HPV in the development of CIN into cervical cancer from an individual evolution perspective to reveal the underlying molecular changes and mechanisms related to HPV‐induced carcinogenesis. We thus suggested that RAB2A could serve as a potential predictive biomarker for the longitudinal monitoring and prognosis of HPV^+^ patients.

Analysis of cervical squamous cell carcinoma shows that human papillomavirus (HPV) integration occurs in more than 80% of cervical cancers,^1^ suggesting the integrations of HPV genomic DNA plays an important role in promoting oncogenesis.^2^ In the present study, we enrolled 101 longitudinal monitoring patients (Figure S1) and collected both HPV^+^ normal cervical epithelium and cervical intraepithelial neoplasia (CIN) tissues to detect HPV integrations (Tables S1 and S2).^3^, ^4^, ^5^ A total of 1041 high‐frequency HPV integrations were thus obtained (Figure S2, Table S3). Among all cervical samples, the overall prevalence rate of HPV integration was 74.8% (157/210). Both the number and the normalized frequency of HPV integrations increased during the early phases of cervical lesions progression, and then significantly decreased during the progression (Figure S3). We next annotated the HPV16 and the HPV18 integration breakpoints and found that breakpoints could appear in any part of the HPV genome, with stage‐specific enrichments during tumour progression (Figures S4–S6). A total of 1041 HPV integrations were annotated in 909 genes from the human genome by ANNOVAR. Several loci showed a significantly high frequency of HPV integrations, including KLF5 (in 50 out of 210 samples), CCAT1 (42/210), FHIT (19/210) and CCDC106 (26/210) (Figure 1A). We investigated the enrichment of HPV breakpoints in CpG islands (Figure 1B). Common fragile regions (CFRs, Figure 1C) and simple repeats (Figure S7) were significantly enriched in both normal and CIN stages, indicating that these integrations may be involved in the early stage of tumour development actively. We also explored the overlap between HPV integrations that were shared between all three stages (Figure 1D). Interestingly, HPV integrations were enriched in different pathways at different stages, indicating a stage‐specific integration paradigm (Figure 1E). In terms of gene structure, HPV integration frequently occurred within the introns of FHIT, CCAT1, CCDC106 and the intergenic region between KLF5/KLF12 (Figure S8). Furthermore, KLF5, LINC00392, CCAT1 and CCDC106 were the most common HPV‐integrated genes in both the normal (HPV^+^) and the CIN stages (Table S4), which were consistent with findings of between‐group comparisons.

**FIGURE 1:**
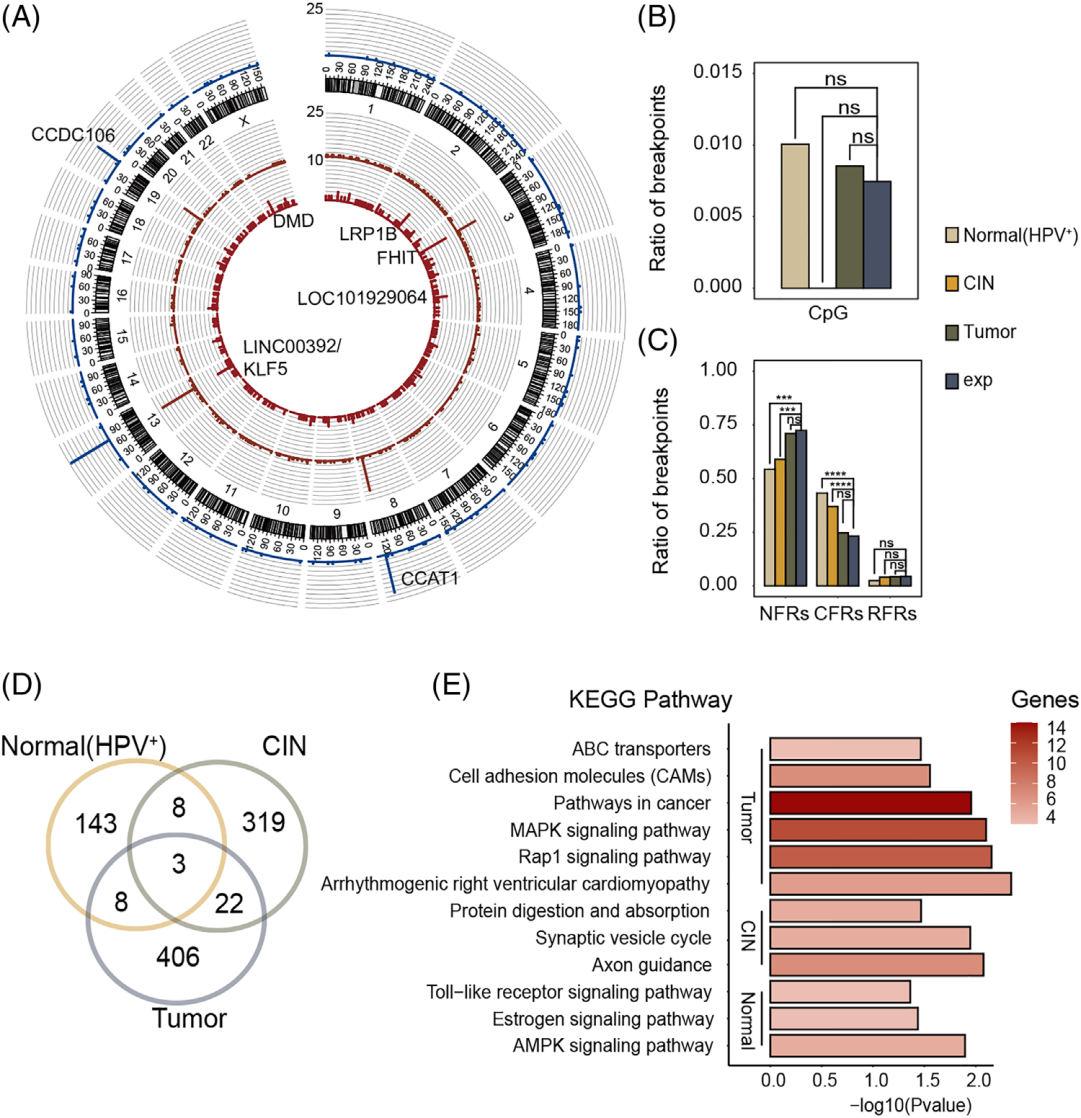
Distribution of breakpoints in the human genome. (A) Hotspot genes of HPV integration at normal (HPV^+^), CIN and tumour stages. From the outer to the inner circle, blue, yellow and red bars represent the frequency of HPV integration. Some loci with high rates of integration are marked. The maximum values are 25, 25 and 10 (statistics were performed for each gene). (B) Enrichment of HPV breakpoints in CpG islands. (C) Enrichment of HPV breakpoints in fragile regions. The following abbreviations were used: NFR, nonfragile regions; CFR, common fragile regions; RFR, rare fragile regions. (D) Venn diagram showing the distribution of HPV integrations. (E) KEGG pathways enrichment for the HPV integrations at different stages

To explore the proteome changes, a subgroup of 20 cervical samples (10 HPV^+^ normal cervical epithelium, and their corresponding CIN samples) with sufficient residual tissues were then selected for data‐independent acquisition‐mass spectrometry. Altogether, we identified 7623 proteins, 555 of which showed significantly different abundances between the two stages (Figures 2A and S9A, Table S5). Between the cervical carcinoma and normal adjacent tissues, 9364 proteins were identified, with 2863 of these were significantly different in abundance (Figures 2B and S9B and Table S6). We used Metascape to identify the pathways that most significantly distinguished CIN from normal (HPV^+^) tissues (Figure 2C), and those that distinguished cervical carcinoma from normal adjacent tissues (Figure 2D). Experimental validation of representative proteins was then performed with parallel reaction monitoring in fresh frozen cervical carcinoma and its adjacent normal tissue. All genes that underwent HPV integration were screened, including 50 protein‐coding genes with ≥2 integration events, and genes coding differentially expressed proteins (Table S7). Our results showed that 21 proteins were significantly regulated in tumour tissues compared with their adjacent tissues, including five downregulated and 16 upregulated proteins (Figure 2E).

**FIGURE 2:**
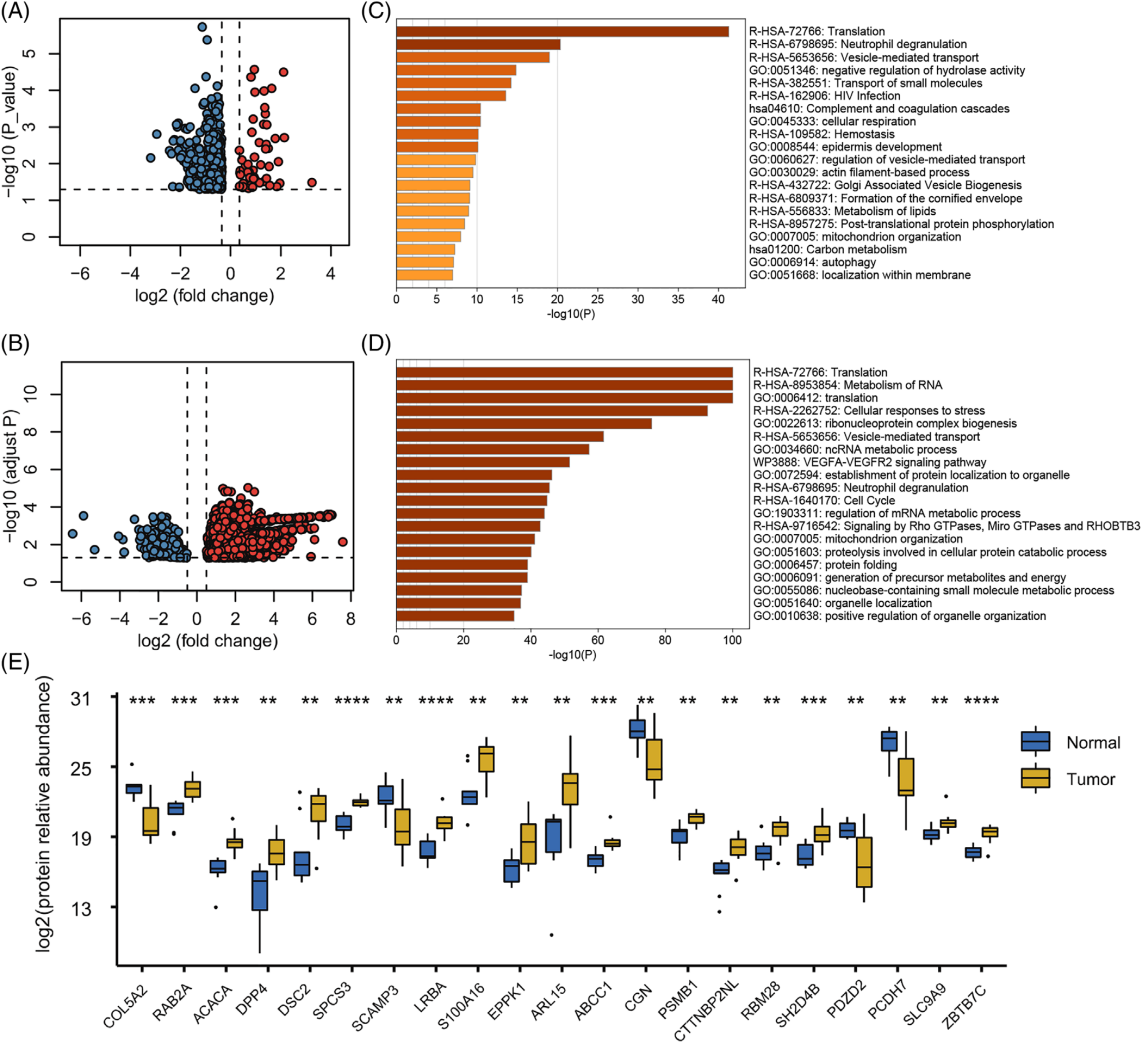
Proteome profiling of cervical specimens at different stages of carcinogenesis. (A) Volcano plots of the proteins with significant abundance differences between CIN and normal (HPV^+^) tissues from the data‐independent acquisition dataset (|log2 (fold‐change)| > 0.35, *p* value ≤ .05). (B) Top 20 clusters of differentially expressed proteins between CIN and normal (HPV^+^) tissues. (C) Volcano plots of the proteins with significant abundance differences between cervical carcinoma and normal adjacent tissues in DIA dataset (|log2 (fold‐change)| > 0.5, adjusted *p* value by *BH* method ≤ .05). (D) Top 20 clusters of differentially expressed proteins between cervical carcinoma and normal adjacent tissues. (E) Expression of 21 selected proteins from the parallel reaction monitoring dataset that were significantly regulated in tumour tissues compared to adjacent normal tissues. Student's *t*‐test was used to compute the significance between normal and tumour samples (unpaired two‐sided Welch's *t*‐test, *p < .05; **p < .01; ***p < .001, ****p < .0001)

To evaluate the effects of HPV integrations on the protein expression during carcinogenesis, we then examined the associations between HPV integrations and proteomic data (Figure S10). We searched for all encoding genes in the vicinity of the integration locus in proteome data and clustered 94 proteins which were differentially expressed in tumour tissues (Figure S11A). We investigated the clinical and prognostic value of the regulated proteins with the TCGA‐CESC cohort using GEPIA2^6^ and found that cervical cancer patients with elevated RAB2A, SPCS3, PLEC, RAB32, NCKAP1, SDF4, RBM28, PLS3 and PFKP had a significantly lower survival (Figures S11B–D and S12). We generated an interaction network^7^ to explore the possible interactions between the 94 differentially expressed proteins and HPV integration events (Figure S13), in which RAB2A was found to interact with RAB32 and SUCLG2. Furthermore, ingenuine pathway analysis identified *cell‐to‐cell signalling and interaction* as the most significantly activated pathway relevant to RAB2A (Figure S14). The expression pattern of RAB2A was examined in the TCGA‐CESC cohort through cBioPortal (Figure S15).^8^ Our results showed that 43 of the 297 patients (14%) displayed RAB2A mRNA alterations.

We found that RAB2A expression was significantly increased in primary cancer compared with its adjacent normal cervical tissue (*p *= .0083, Figure S16). Because RAB2A basal expression in the C33A and ME180 cell lines was higher than that in the SiHa and HeLa cell lines (Figure 3A and B), C33A and ME180 cells were therefore transfected with RAB2A siRNA (Figure 3C and D) and showed significantly decreased scratch wound healing and lower migration abilities (Table S8; Figures 3E–G and S17). Our results also showed that the overexpression of RAB2A significantly promoted cell migration and invasion (Table S9; Figures 4A–D and S18 and S19). We also observed that the expression of E‐cadherin decreased, while N‐cadherin and Snail‐1 had an increased expression in SiHa and HeLa cells overexpressing RAB2A. The opposite trend was observed for RAB2A knockdown ME180 cells (Figure 4E and F). These results indicated that RAB2A may affect epithelial‐to‐mesenchymal transition induction. The animal experiments showed that the overexpression of RAB2A promoted tumour growth and metastasis in nude mice (*p *< .05). Furthermore, as shown in Figure 4G, liver metastasis was found in one out of five experimental mice, whereas no metastasis occurred among the controls.

**FIGURE 3:**
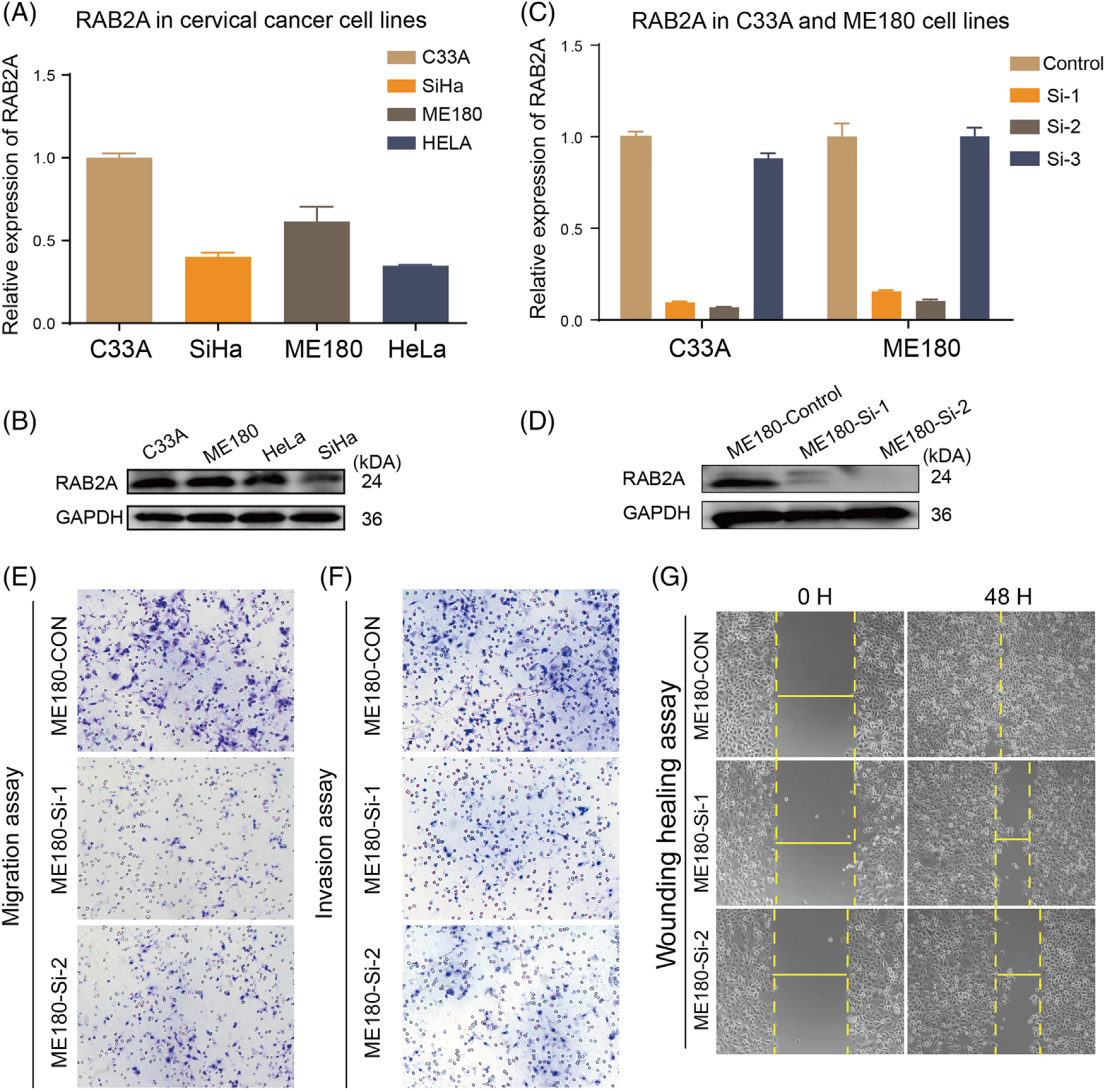
The downregulation of RAB2A suppresses the migration and invasion of cervical cells. (A, B) The basal level of RAB2A in cervical cancer cell lines was measured by qPCR and western blot. (C, D) The efficiency of siRNA in C33A and ME180 cells was assessed by qPCR and western blot. (E, F) Transwell migration and invasion assays performed with ME180 cells. (G) Wound healing scratches imaged immediately and 48 h after the initial scratch to quantify the relative migration

**FIGURE 4:**
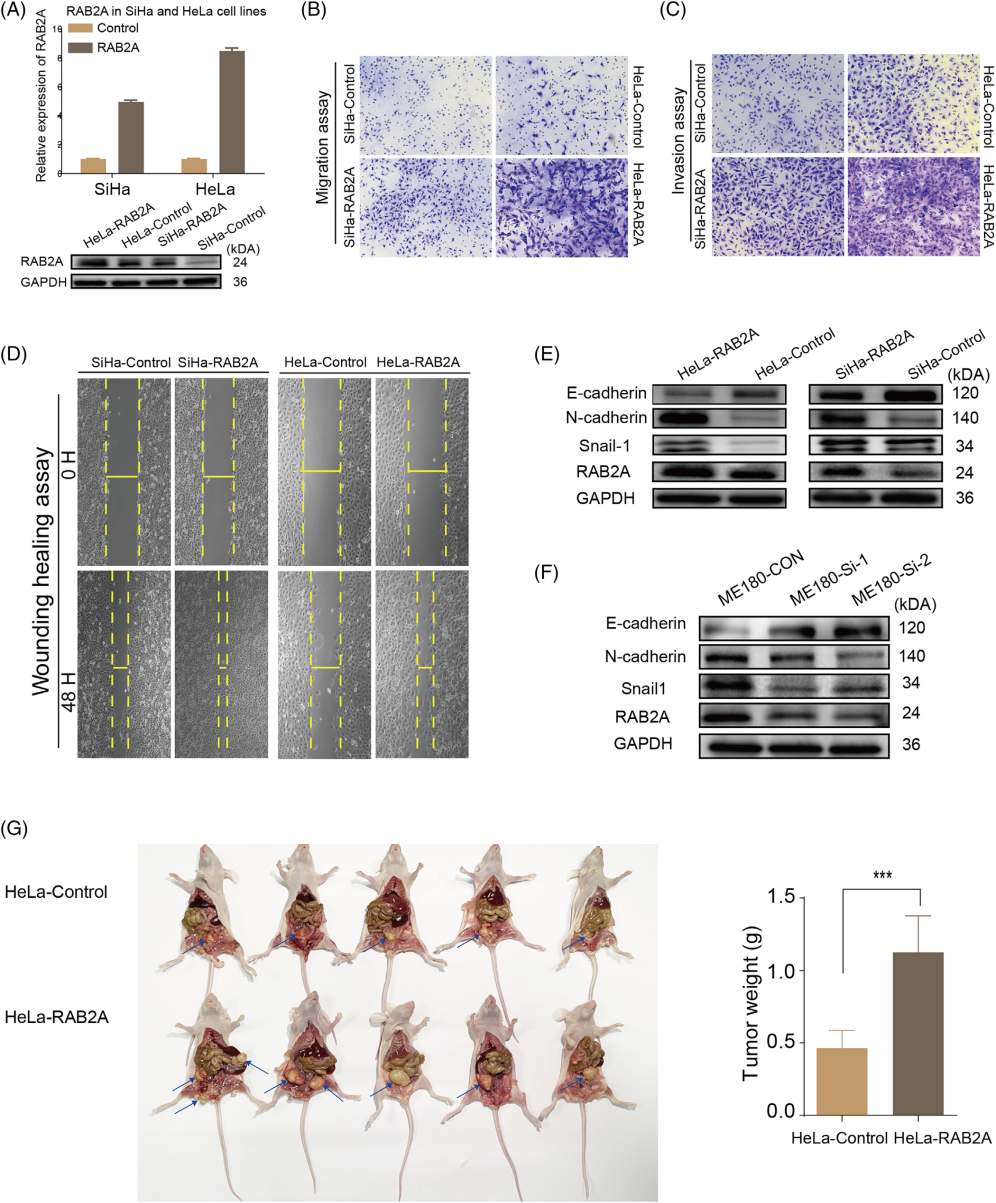
The upregulation of RAB2A promotes the migration and invasion of SiHa and HeLa cells. (A) Lentiviral transfection efficiency measured by qPCR and Western blot. (B, C) Transwell migration and invasion assays performed with SiHa and HeLa cells. (D) Wound healing scratches imaged immediately and 48 h after the initial scratch to quantify the relative migration. (E) Effects of RAB2A overexpression on EMT marker in SiHa and HeLa cells. (F) Effects of RAB2A knockdown on EMT marker in ME180 cells. (G) HeLa cells stably overexpressing RAB2A were inoculated into the cervix of female nude mice to generate orthotopic mice models. The arrows indicate the tumours' locations

In conclusion, RAB2A may serve as a potential predictive biomarker for longitudinal monitoring in HPV^+^ patients. Future investigations of our proteomics and HPV integration datasets could provide further insights into cervical cancer development.

## COMPETING INTERESTS

The authors declare that they have no competing interests.

## Supporting information

Supporting information

Supporting information
