# Supplementary material for: RAB2A promotes cervical cancer progression as revealed by comprehensive analysis of HPV integration and proteome in longitudinal cervical samples
Source: Clin Transl Med. 2022 Mar 28;12(3):e767. doi: 10.1002/ctm2.767 (PMC8958348; doi:10.1002/ctm2.767)
Supplement: Supplementary file 2 — Supporting information [file CTM2-12-e767-s002.docx]

## Supplementary Methods

**Study design and patient cohort**

This study was approved by the Ethical Committee of Tongji Hospital of Tongji Medical College of Huazhong University of Science and Technology. All participants were recruited either from Tongji Hospital of Tongji Medical College of Huazhong University of Science and Technology or from the Qilu Hospital of Shandong University. All patients gave informed consent. Enrollment criteria included: female patients, aged >18 years and sexually active, who had had at least 2 consecutive HPV positive tests and colposcopy examinations with pathological diagnosis; the pathological diagnosis of the cervical specimens from the first colposcopy biopsy that showed no cervical precancerous lesions, and the pathological diagnosis of the cervical specimens in the follow-up examination that was either cervical intraepithelial neoplasia (CIN) or cervical cancer; the interval between initial and follow-up visits that was greater than three months. Follow-up data and statistics were recorded for all patients up to May 31st, 2019.

**Specimen acquisition**

The pathological diagnoses of all cervical specimens were reviewed by experienced pathologists. To obtain biopsy-scale samples, we used a needle to collect tissue cylinders (diameter: 1 mm, length: ~3 mm, wet weight: ~2 mg) at the confirmed lesions of formalin-fixed paraffin-embedded (FFPE) specimens. Since cervical specimens from the first colposcopy biopsy showed no cervical precancerous lesions, the squamous epithelium was punched for analysis.

**DNA extraction and high­throughput viral integration detection**

High-quality genomic DNA was extracted and purified from FFPE cervical samples using GeneRead DNA FFPE Kit (#180134, Qiagen) following the manufacture’s instructions. The pathological diagnosis for all samples is available in Table S1. The hybridization and sequencing process was carried out according to MyGenostics GenCap Target Enrichment Protocol (GenCap Enrichment, MyGenostics, USA) and the instructions from Illumina (Illumina Inc., San Diego, CA). The full construction and operational details were as previously reported.

A total of 85 cervical specimens, derived from 42 patients, generated enough DNA (≥ 3 µg, with a concentration ≥20 ng/µL, and an apparent main band upon electrophoresis) to be analyzed by high­throughput sequencing. The reference genomes used for alignment were hg38 for human (UCSC) and 83 types of HPV (Table S2). ANNOVAR was used for the annotation of HPV integration sites. Every integration site was annotated to 1 or 2 nearby genes within 500kb upstream or downstream. To enable comparisons across samples, the supporting read numbers of HPV integrations were normalized by the sequencing depth and the capture efficiency.

**Peptide extraction and quantitative mass spectrometry**

We prepared cervical samples for proteomics analysis as previously described. Liquid chromatography with tandem mass spectrometry (LC-MS/MS) with data-independent acquisition (DIA) was performed on the DIONEX UltiMate 3000 nano System coupled with the Q Exactive HF hybrid Quadrupole-Orbitrap (Thermo Fisher Scientific™, San Jose, USA). Parallel reaction monitoring (PRM) -based targeted mass spectrometry was used to validate selected proteins. The Q Exactive HF hybrid Quadrupole-Orbitrap was operated in the MS/MS mode with time-scheduled acquisition for 50 peptides in a +/− 5 min retention time window.

**DIA mass spectrometry**

The LC-MS/MS analysis was performed on the DIONEX UltiMate 3000 nano System coupled with the Q Exactive HF hybrid Quadrupole-Orbitrap (Thermo Fisher Scientific™, San Jose, USA). Biognosys-11 iRT peptides (Biognosys, Schlieren, CH) were spiked into the peptide samples at the final concentration of 10% before MS injection for RT calibration. For all samples, peptides were separated at 300 nL/min, for 90 min, in a 3–28% linear gradient (buffer A: 2% ACN, 0.1% FA; buffer B: 98% ACN, 0.1% FA). Eluted peptides were ionized into a Q-Exactive HF mass spectrometer (Thermo Fisher Scientific, San Jose, USA). A full MS scan was acquired analyzing 390–1,010 m/z at resolution 60,000 (at m/z 200) in the Orbitrap using an AGC target value of 3E6 charges and maximumion accumulation time 80 ms. After the MS scan, 24 MS/MS scans were acquired, each with a 30,000 resolution at m/z 200, AGC target 1E6 charges, and maximum ion injection time of 55 ms. DIA MS data search was based on the DPHL library and performed by DIA-NN (Version 1.6.0) according to the official manual. The precursor false discovery rate was set to 0.01. The peptide matrices were converted to protein matrixed by ProteomeExpert.

**Validation of representative proteins using PRM**

PRM quantification strategy was used to further validate a subset of proteins. Biognosys-11 iRT peptides (Biognosys) were spiked into the peptide samples at the final concentration of 10% before MS injection for RT calibration. Peptides were separated at 300 nl/min along a 30 min, 7–35% linear LC gradient (buffer A: 2% ACN, 0.1% FA; buffer B: 98% ACN, 0.1% FA). The Q Exactive HF hybrid Quadrupole-Orbitrap (Thermo Fisher Scientific™, San Jose, USA) was operated in the MS/MS mode with time-scheduled acquisition for 50 peptides in a +/− 5 min retention time window. The isolation window was set at 1.6 m/z. The full MS mode was measured at a resolution of 60,000 (at m/z of 200) in the Orbitrap, with an AGC target value of 3E6 and maximum ion accumulation time of 50 ms. MS/MS spectra were acquired at resolution 30,000 (at m/z of 200) in the Orbitrap using AGC target value of 2E5 and maximum ion accumulation time of 100 ms.

**Data analysis and pathway enrichment analysis**

A two-sided unpaired Welch’s t-test, followed by Benjamini and Hochberg correction, was used to compute *P*-values in order to compare cervical carcinoma with its normal adjacent tissue (HPV^+^). Similarly, a paired Student's t-test was performed to compare CIN with normal (HPV^+^) tissues. All these analyses were done in R. Network and pathway analysis tools were then used to identify the most highly affected pathways. The top enriched pathways were identified by Metascape. Ingenuine pathway analysis (IPA) returned the most significantly enriched pathways. The *P*-value was calculated by right-tailed Fisher’s Exact Test. And the overall activation or inhibition states of the enriched pathways were predicted using the z-score.

**Immunohistochemistry**

A total of 44 cervical cancer and 25 normal cervix samples were obtained from The Biobank of Patients With Gynecologic Neoplasms (NCT01267851). FFPE sections (4 μm) were subjected to immunohistochemistry (IHC) staining using an IHC Kit (#SP-9001, Origen). The slides were incubated overnight at 4°C with an antibody against RAB2A (#ab154729, Abcam, 1:100). DAB (#G1212-200T, Servicebo) was used for staining detection. For each sample, the immunoreactivity score (0-12) was calculated by multiplying the staining intensity score (0-3) by the percentage of staining positive cells (0-4).

**Cell culture**

SiHa, HeLa, ME180, and C33A cell lines were all obtained from American Tissue Type Culture Collection (ATCC, Manassas, VA) and cultured following ATCC guidelines (DMEM (Gibco, USA) supplemented with 10% FBS (Hyclone, #SH30406.05) and 100 U/ml penicillin/streptomycin). All cell lines were cultured in an incubator with 5% CO_2_, at 37 °C.

**siRNA, lentiviral plasmid construction and transfection**

Three siRNA sequences targeting the human RAB2A gene and control siRNA were used for transfection in ME180 and C33A cells. The siRNAs were purchased from Riobio, Inc. (Guangzhou, China) and their sequences are listed in Table S3. To construct the RAB2A expression vector, the fragments encompassing the RAB2A sequence (NCBI reference sequence NM_002865) were chemically synthetized and then cloned into the AgeI sites in a lentiviral vector (GV358) (Genechem, Shanghai). The vector was then co-transfected into SiHa and HeLa cells with the lentiviral genomic plasmids. Puromycin (0.4 µg/ml, #A1113803, Gibco) was used to maintain the selective pressure for 2 weeks. All experiments were performed according to the manufacturer’s instructions.

**RNA Extraction, PCR, and quantitative real-time PCR (qPCR)**

Total RNA was extracted from cells using TRIzol reagent (#15596026, Invitrogen). HiScript II Q RT SuperMix for qPCR (R223-01, Vazyme) was then used to synthesize cDNA. The levels of RAB2A mRNAs were determined using qPCR on Bio-Rad CFX96 Real-Time System manager (C1000 Thermal Cycler), with GAPDH as internal control. The specific primer sequences of targeted genes are reported in Table S4.

**Western Blot Analysis**

Cells were lysed in a radioimmunoprecipitation assay (RIPA) buffer (#P0013B, Beyotime) with a 1% protease inhibitor cocktail (#04693132001, Roche). Approximately 30 μg proteins were loaded onto SDS-PAGE gel (#8012011, BioSci), and transferred to a PVDF membrane (#10600023, GE) at 300 mA for 2 hours. The PVDF membrane was blocked with 5% BSA (bovine serum albumin) for 1 hours at room temperature, and then incubated overnight, at 4°C, with anti-RAB2A (#A11634, Abclonal, 1:1000), anti-GAPDH (#A19056, ABclonal, 1:1000), anti-E-cadherin (#A11492, ABclonal, 1:1000), anti-N-cadherin (#A19083, ABclonal, 1:1000), and anti-Snail (#A11794, ABclonal,1:1000) antibodies. After washing, the membrane was incubated with HRP Goat anti-rabbit IgG (H+L) (#AS014, ABclonal,1:5000) at room temperature for 60 minutes, and washed three times in tris-buffered saline-Tween (TBST). The PVDF membrane was performed using western bright ECL HRP substrate (#K-12045-C20, Advansta) and analyzed by Image Lab (version 4.1). All assays were performed in triplicate.

**Transwell migration and invasion assays**

Cell migration and invasion assays were performed using Transwell chambers (8 μm pores, Corning) pre-coated without (for migration assay) or with (for invasion assay) Matrigel (#356234, BD Biosciences) according to the standard protocols. First, 5×10^4^ cells suspended in 200 μl of serum-free medium were plated in the upper chambers, whereas 500 μl of medium supplemented with 10% FBS was placed in the lower chambers. Following a 24 hours incubation, the cells on the upper surface of the membrane filter were fixed with 4% paraformaldehyde for 10 minutes and then stained with 0.1% crystal violet for 30 minutes. Finally, the upper chamber biofilm was cut off for further analysis.

**Wound Healing Assay**

To perform a wound-healing assay, horizontal and vertical scratches were made using pipette tips in the six-well plates. The cells were then subjected to serum-free medium for 48 hours. Subsequent cell migration was observed by microscopy at 0 and 48 hours.

**In vivo xenograft tumor models**

Six weeks old BALB/c-nude female mice were purchased from Beijing HFK Bioscience Co. Ltd. All animal procedures were approved by the Ethics Committee for Animal Experiments of the Hubei province. Cervical cancer cells were collected at the logarithmic growth stage, and then subcutaneously injected into the mice cervices. At the end of the experiments all the mice were euthanized, and a photo of each tumor was taken in situ. Tumor tissues were then removed and weighed after a complete necropsy.


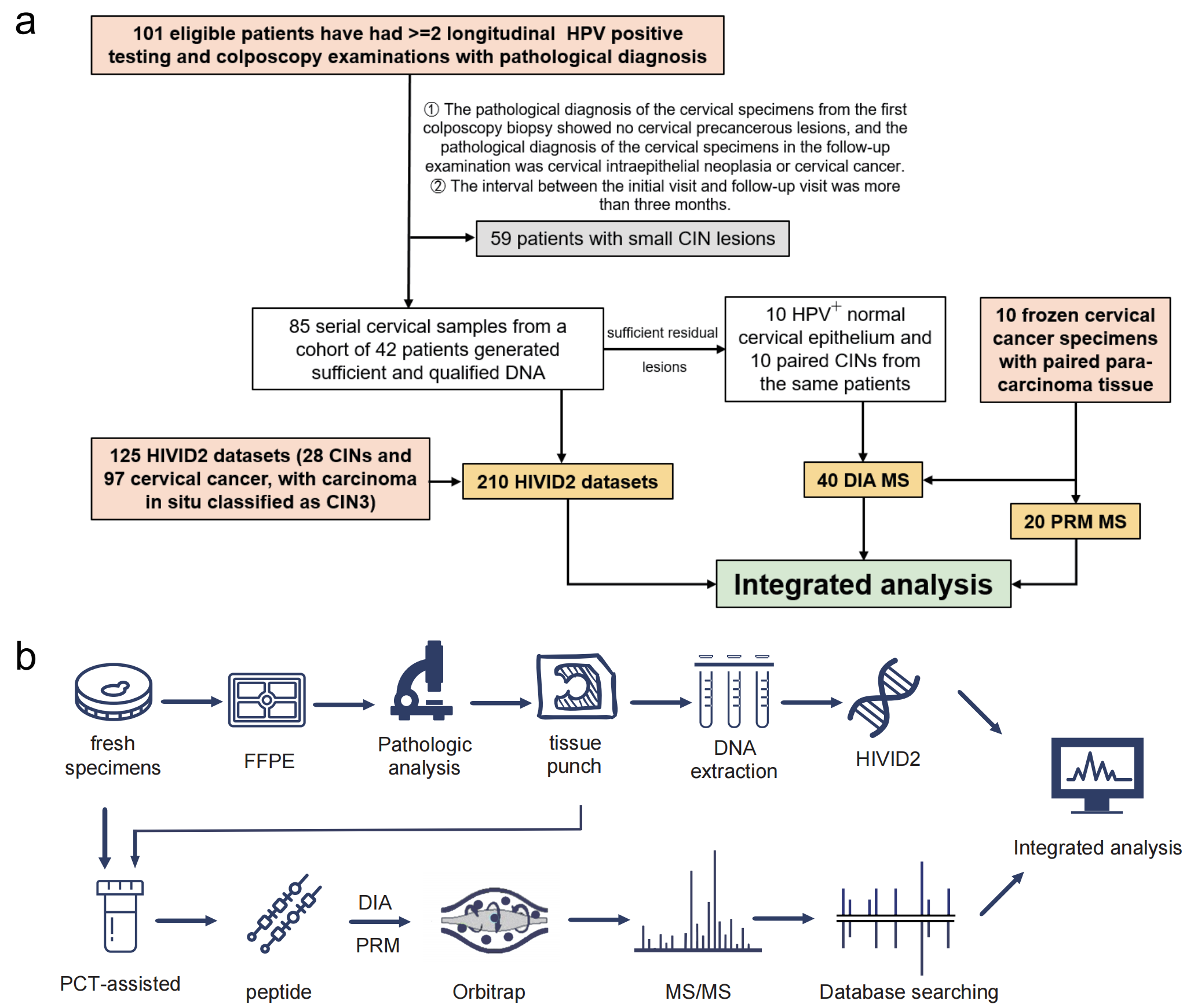


## Figure S1 Overview of the study design, and landscape of HPV integrations.

a. Patient inclusion criteria and withdrawals, with the resulting number of cervix samples included in the study. b. Experimental study design. The following abbreviations were used: FFPE, formalin-fixed paraffin-embedded tissue; PCT, pressure cycling technology; DIA, data-independent acquisition; PRM, parallel reaction monitoring; MS/MS, tandem mass spectrometry.


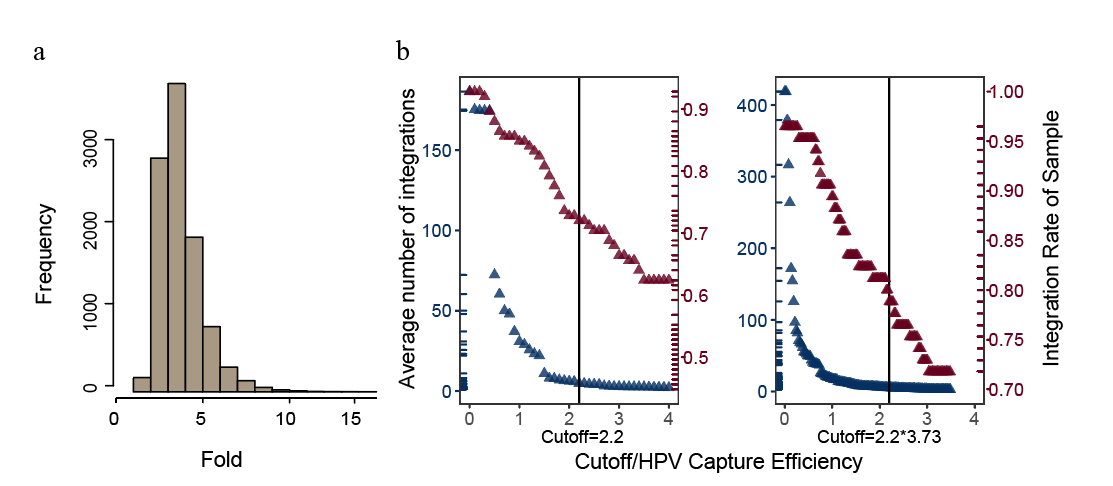


## Figure S2 Evaluation of HPV capture efficiency and threshold selection.

(a) Distribution of fold changes of HPV capture efficiency. (b) The trend of the average integration number and the integration rate in SRA (left, HPV capture efficiency is 1) and Infect (right, HPV capture efficiency is 3.73) after increasing the threashold. The blue triangle and the red triangle represent The average number of integrations and the integration rate are shown in blue and red, respectively.


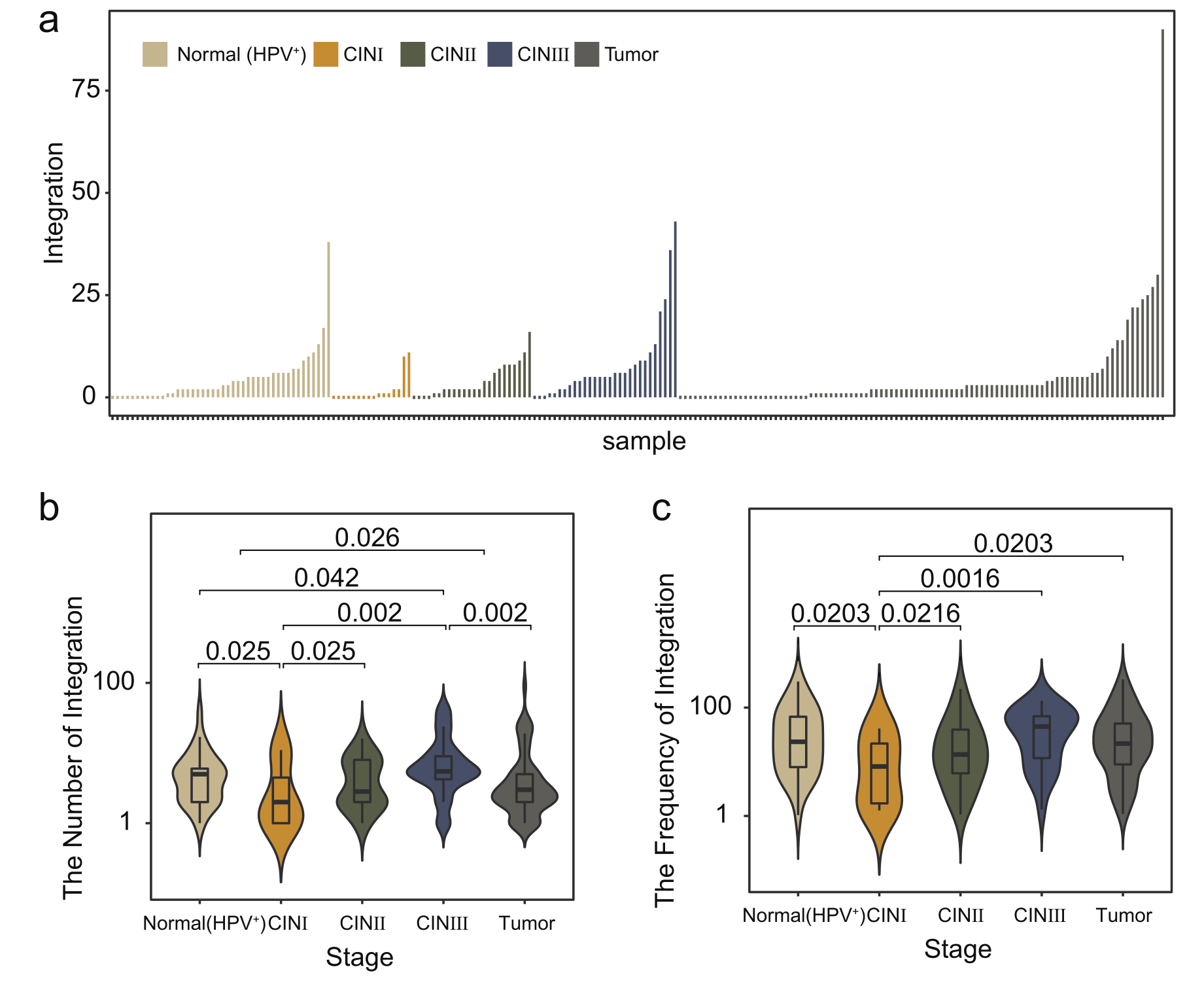


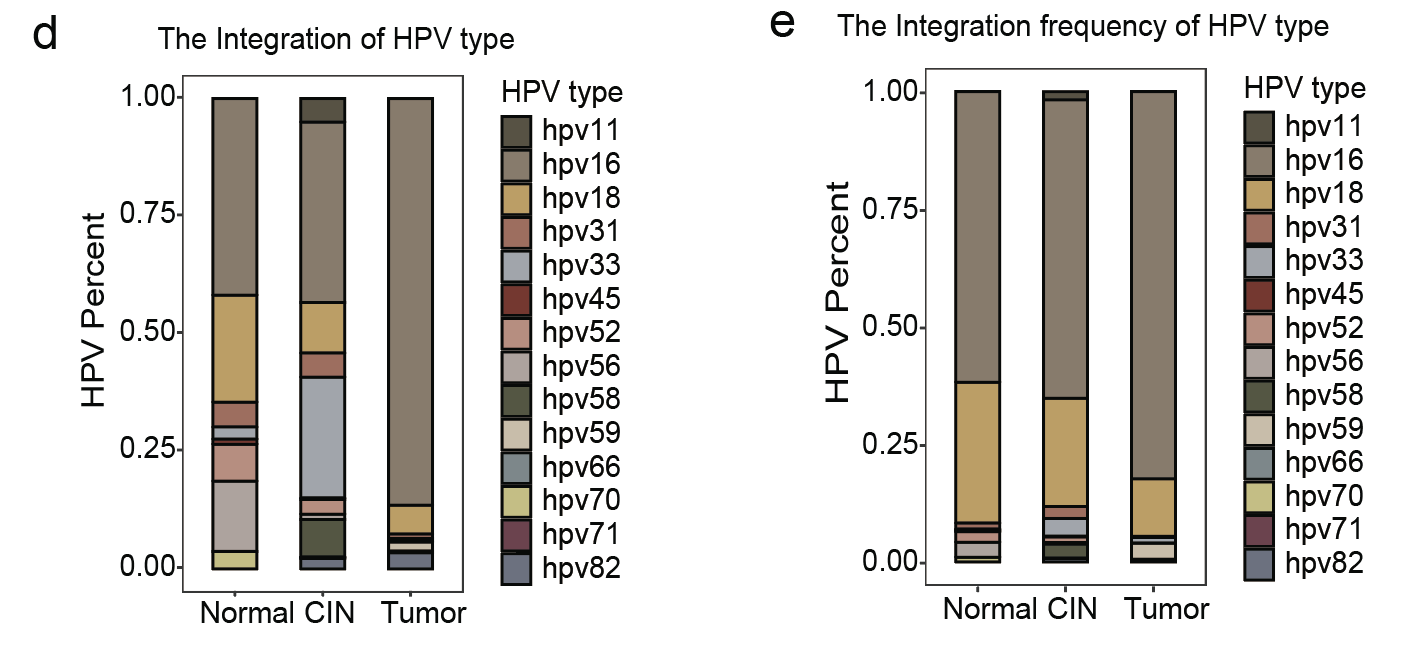


## Figure S3 Overview of the HPV integrations.

a. The distribution of HPV integration numbers at different stages: normal (HPV+), CINⅠ, CINⅡ, CINⅢ, and tumor stage. b. Distribution of the HPV integration numbers (two sided Wilcoxon test, P-value adjusted by Benjamini-Hochberg (BH)). c. Distribution of the normalized HPV integration frequencies (two sided Wilcoxon test, P-value adjusted by BH). d. The proportion of integrated HPV types. e. The proportion of the normalized frequencies of integrated HPV types.


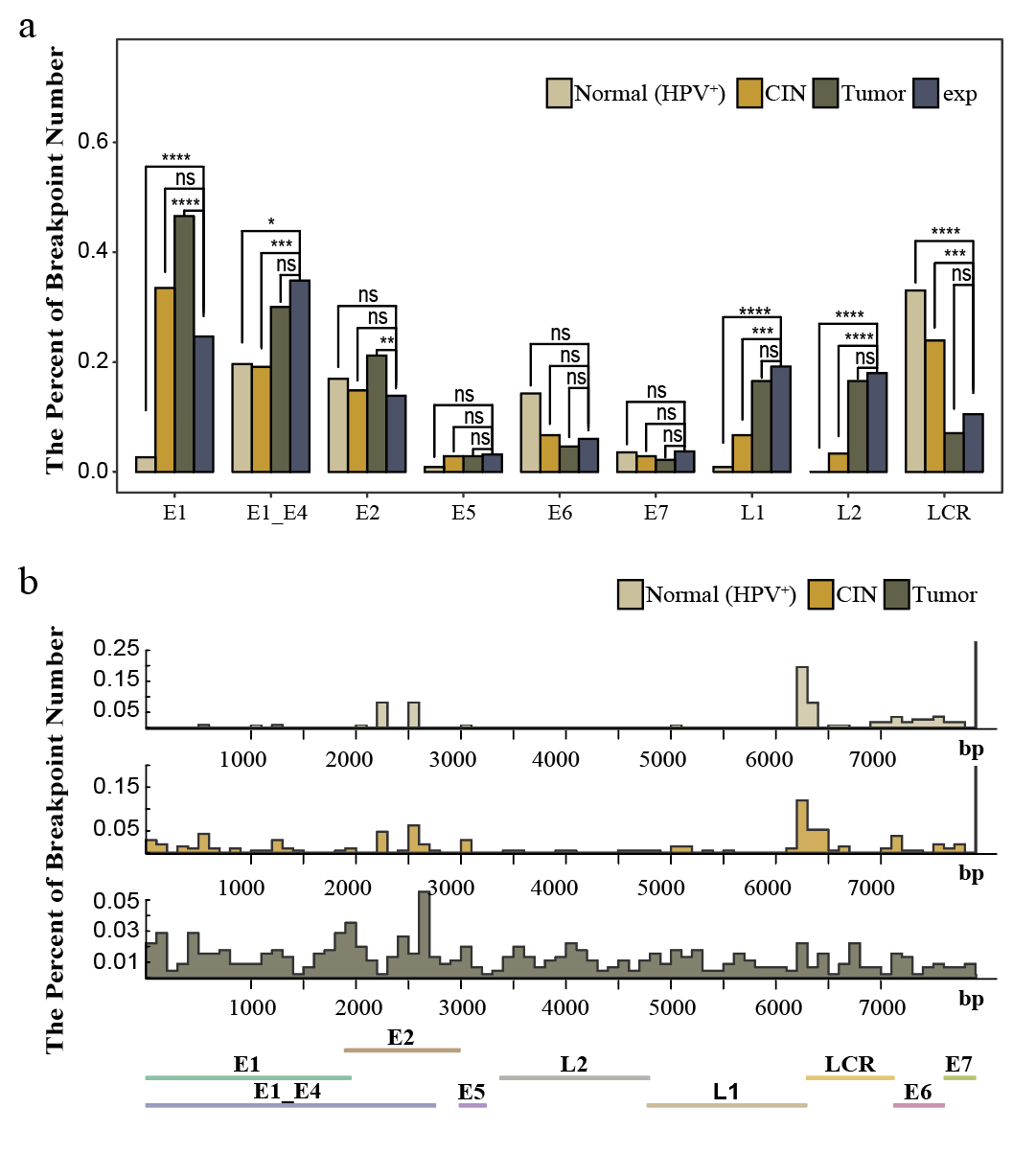


## Figure S4 Distribution of HPV integration sites at the HPV16 genome level.

(a) Enrichment of integrations in the genes or bicistronic RNA of HPV16 at Normal (HPV+), CIN, and Tumor (the percentage of integrations at each stage was calculated, Fisher's exact test, two-sided). (b) Distribution of integrations in the HPV16 complete genome at Normal (HPV+), CIN, and Tumor stages (statistics were performed in units of 100bp bins).


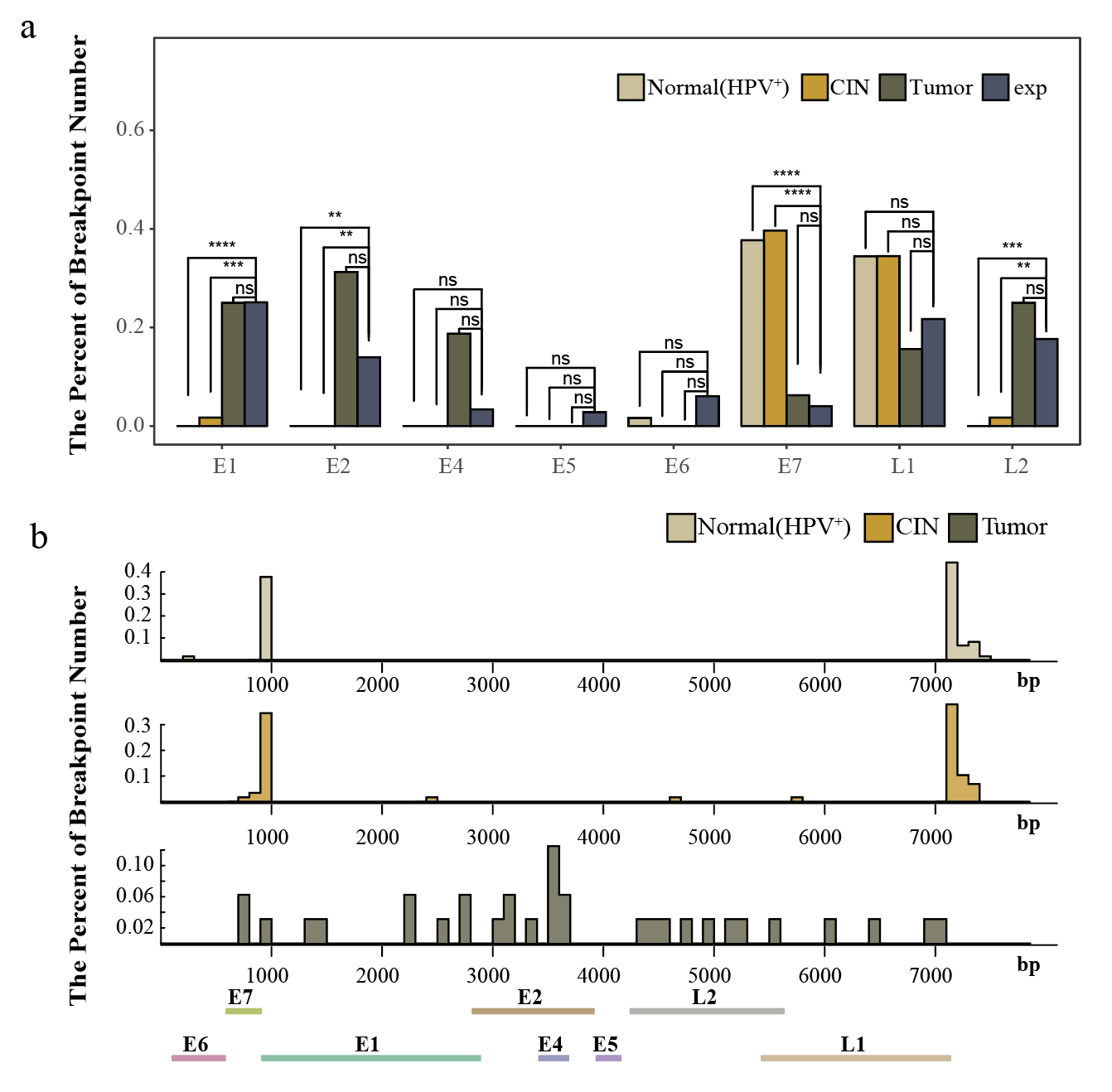


## Figure S5 Distribution of HPV integration sites at the HPV18 genome level.

(a) Enrichment of integrations in the genes of HPV18 at Normal (HPV+), CIN, and Tumor (the percentage of integrations at each stage was calculated respectively, Fisher's exact test, two-sided). (b) Distribution of integration in the HPV18 complete genome at Normal (HPV+), CIN, and Tumor stages (statistics were performed in units of 100bp bins).


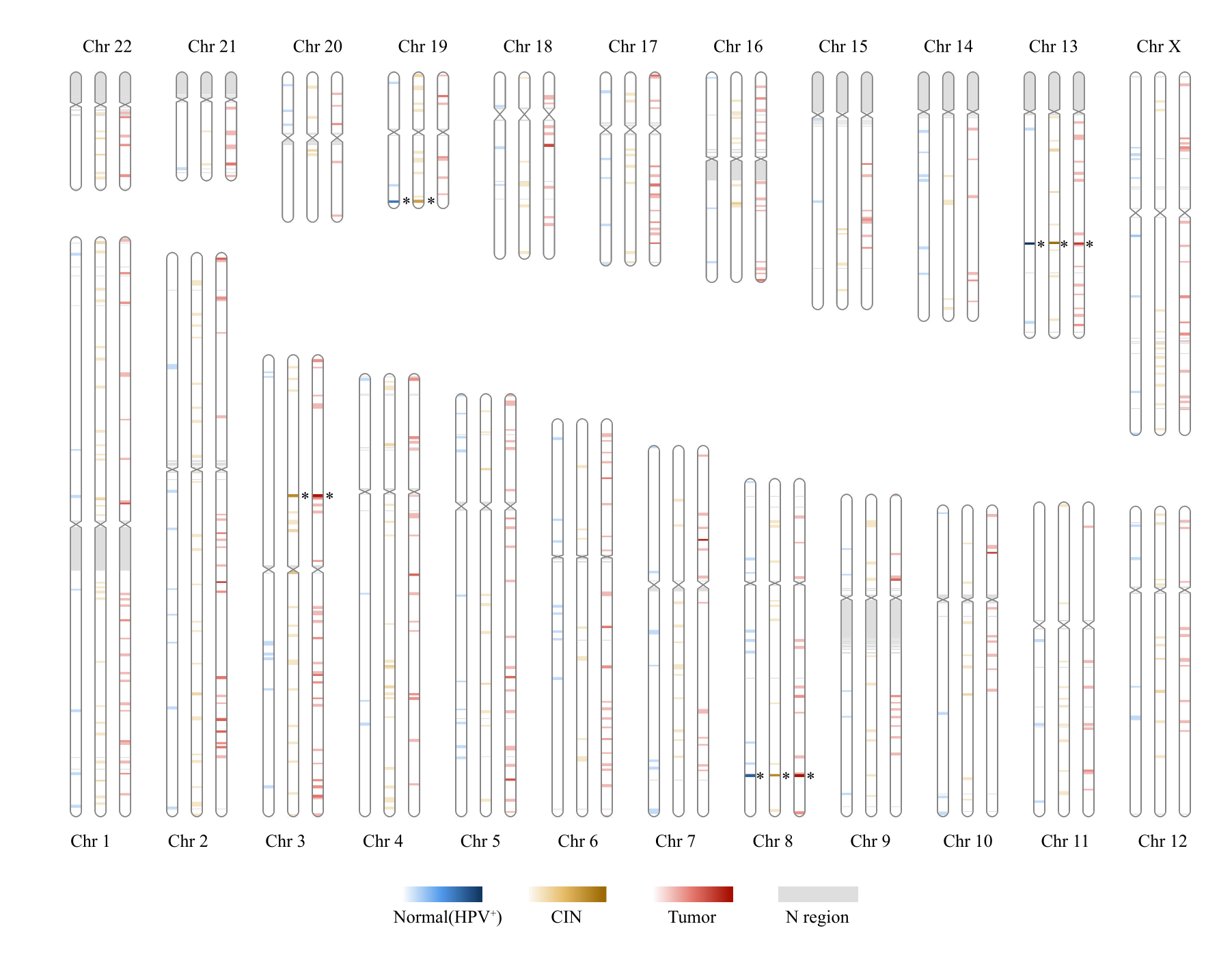


## Figure S6 Distribution of the HPV integration regions in each chromosome.

The blue, yellow, and red bars highlight the integrations occurring at the Normal (HPV+), CIN, and Tumor stage. Each chromosome is divided into 1Mb windows to count the number of integrations. Darker colors indicate greater numbers of integrations. When calculating the expected number, it is assumed that all integrations are randomly distributed across the genome without the N region. The enriched regions (marked by “*”) have p-values<0.05, which were calculated by Fisher's exact test for each stage (Normal(HPV+), CIN, and Tumor).


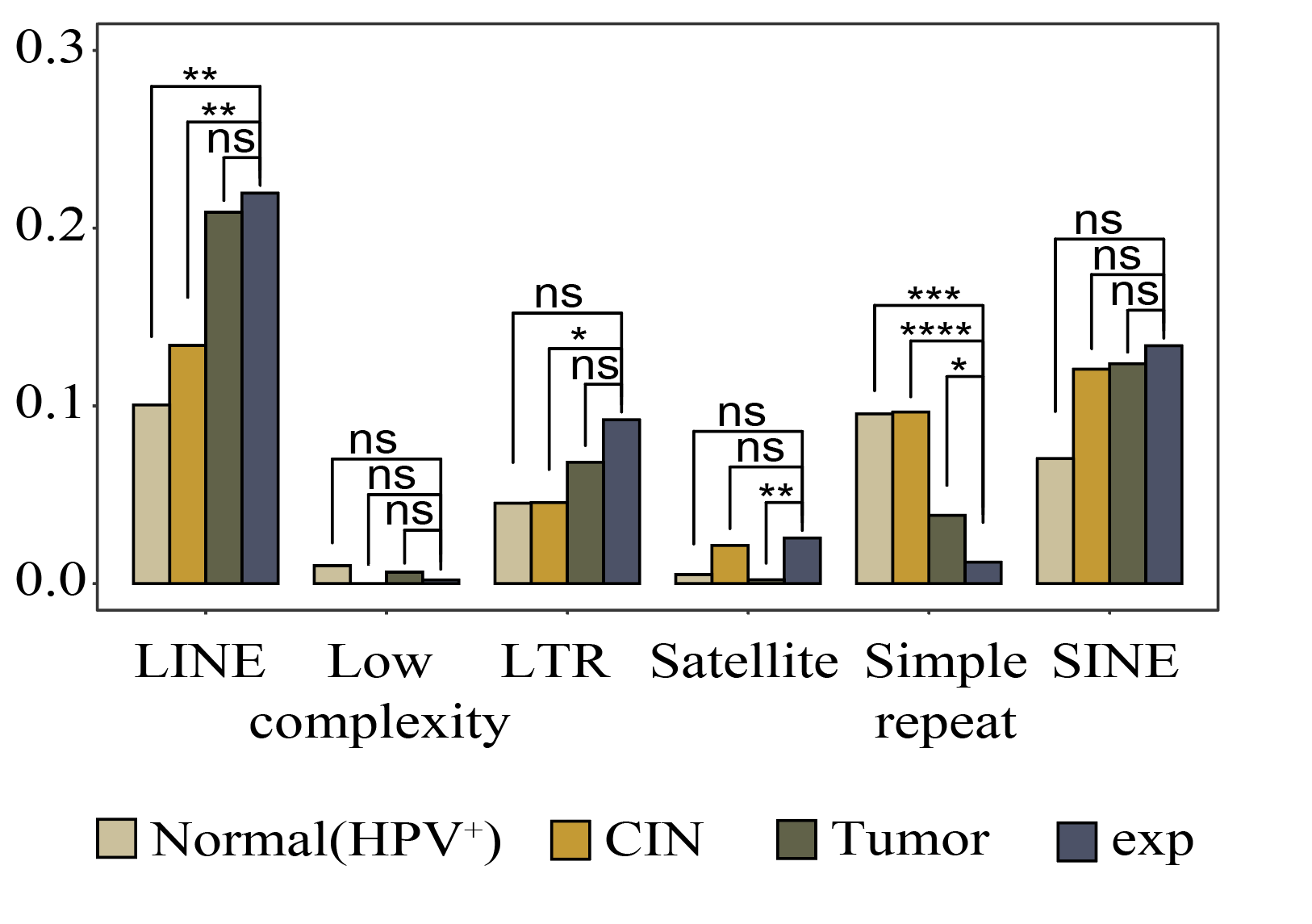


Figure S7 HPV integration and enrichment in the repeated region (Fisher's exact test, two sided).


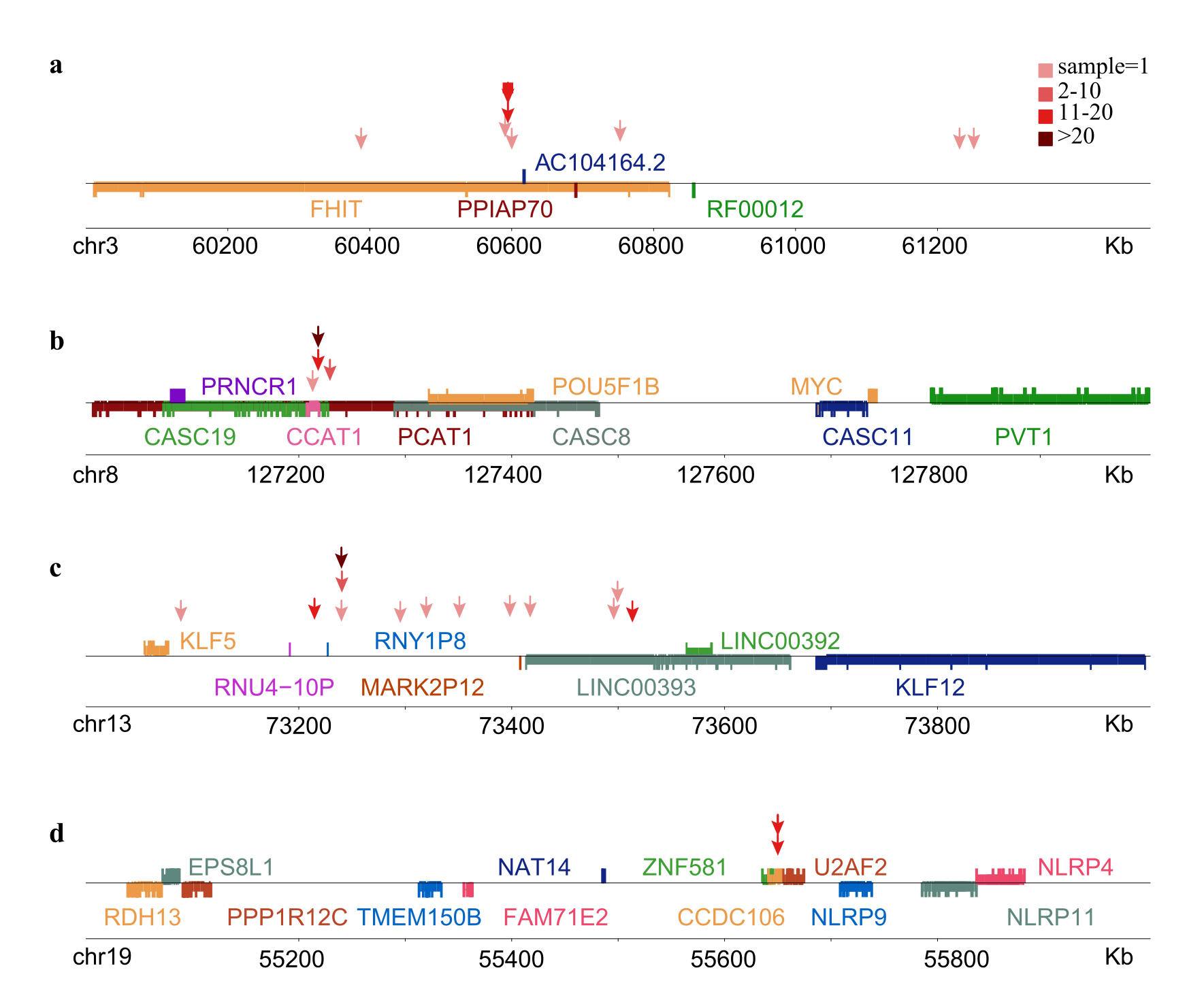


## Figure S8 Hotspots where HPV integration most frequently occurs.

(a) FHIT, (b) CCAT1, (c) KLF5/KLF12, and (d) CCDC106 genes.


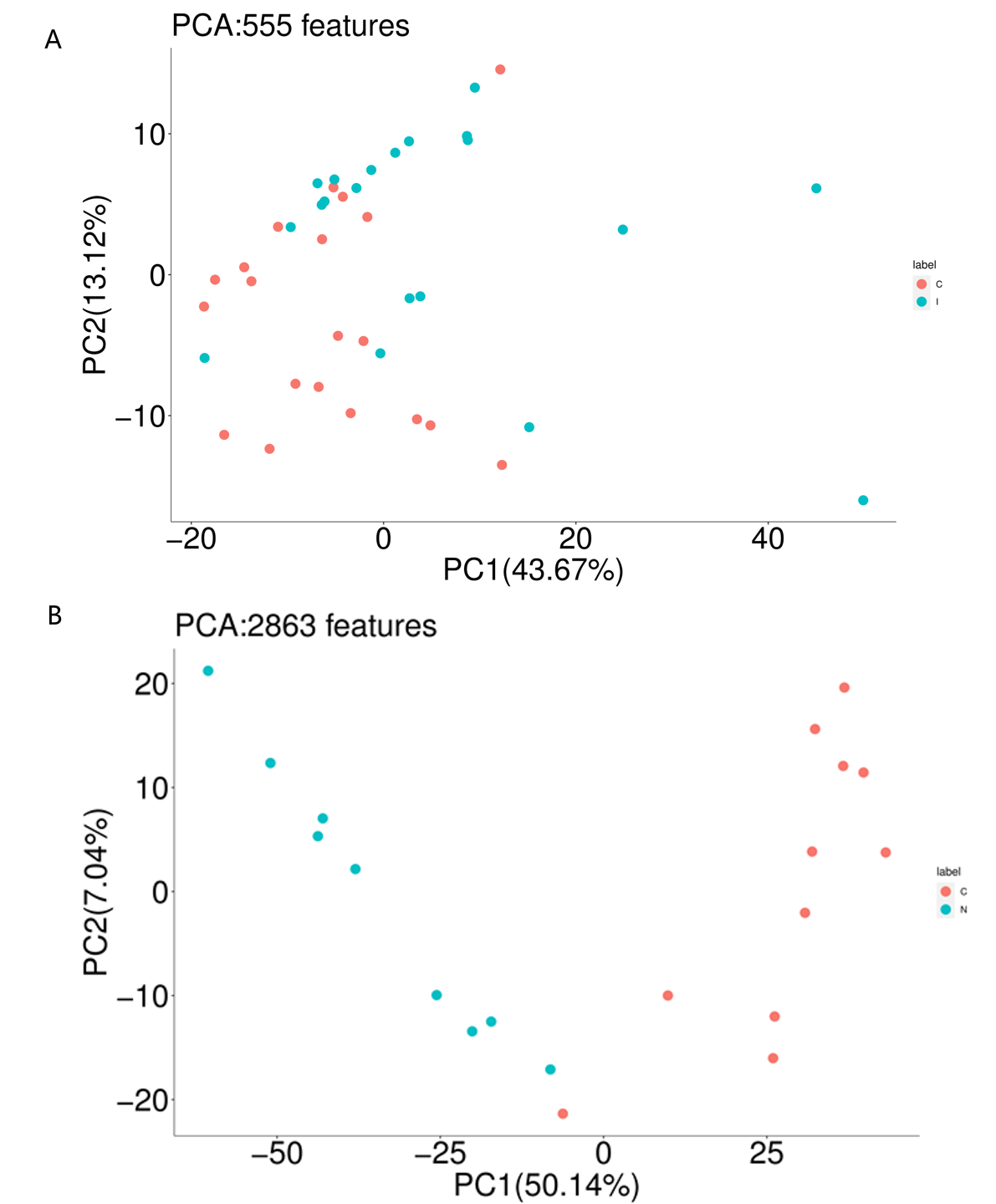


## **Figure S9** Unsupervised PCA analysis.

(a) Unsupervised PCA analysis based on the 555 most regulated protein expression in CIN vs the normal (HPV+) group.

(b) Unsupervised PCA analysis based on the 2,863 most regulated protein expression in cervical carcinoma vs the normal adjacent tissues,


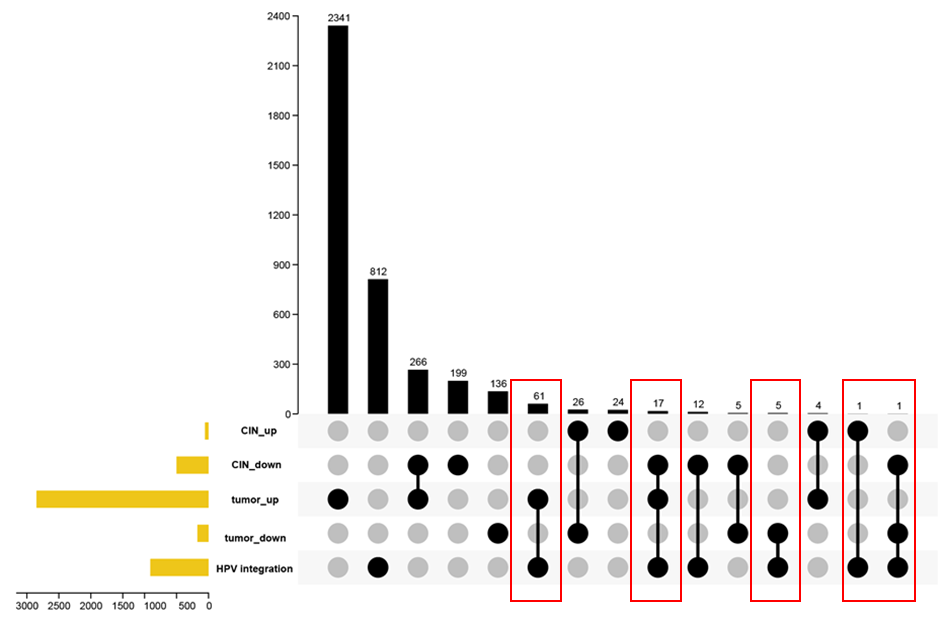


Figure S10 Visualization of tissue intersections of significantly regulated proteins and HPV breakpoints. The number above the bar showed the overlaps of differentially expressed peptides and proteins between HIVID and proteomic data.


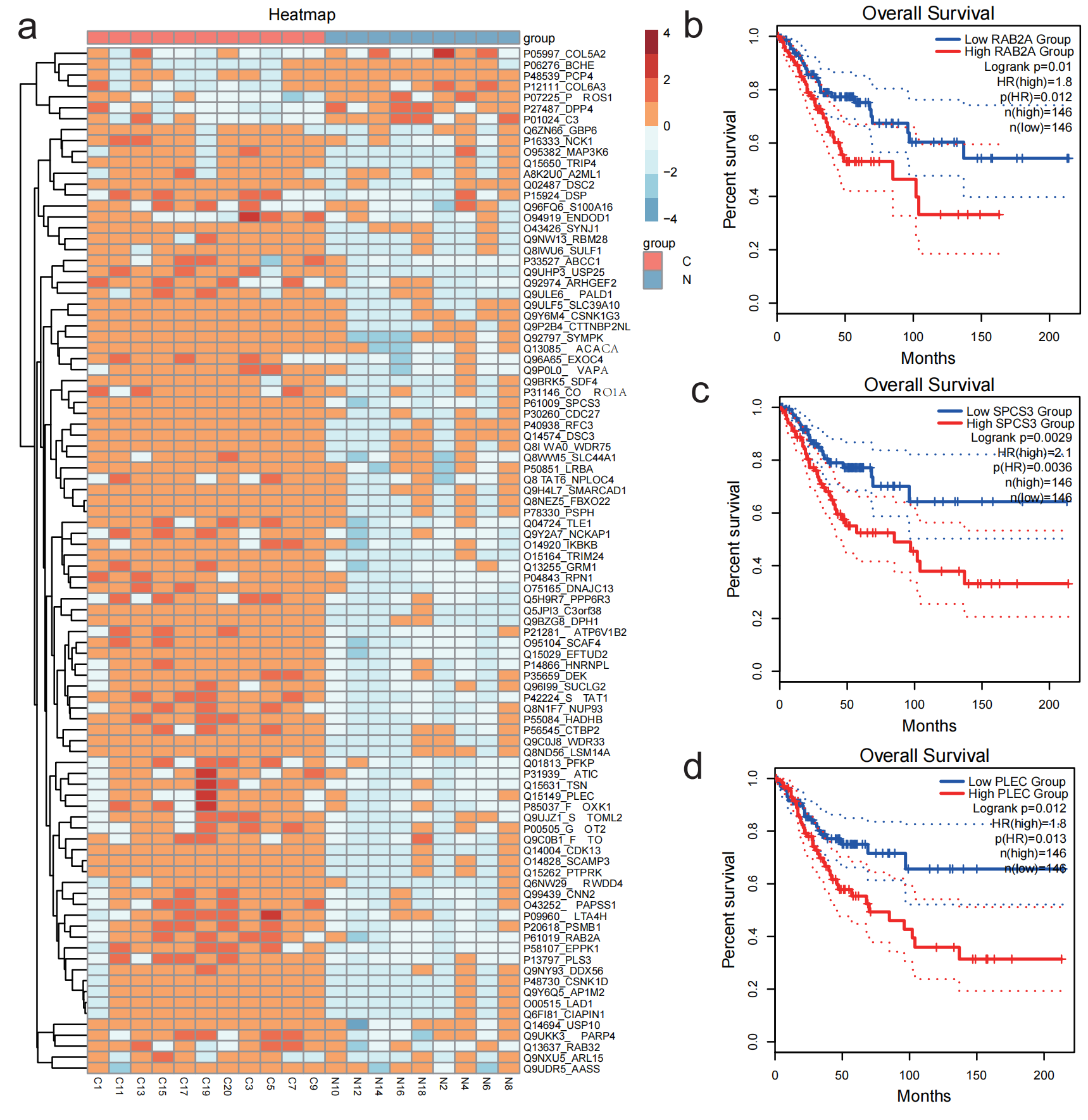


## **Figure S11 Integrated analysis of HPV integrations and proteome data.**

a. Cluster analysis of 94 differentially expressed proteins with HPV integration events. b-d. Kaplan-Meier survival curve analysis based on the TCGA CESC database for RAB2A (b), SPCS3 (c), and PLEC (d).


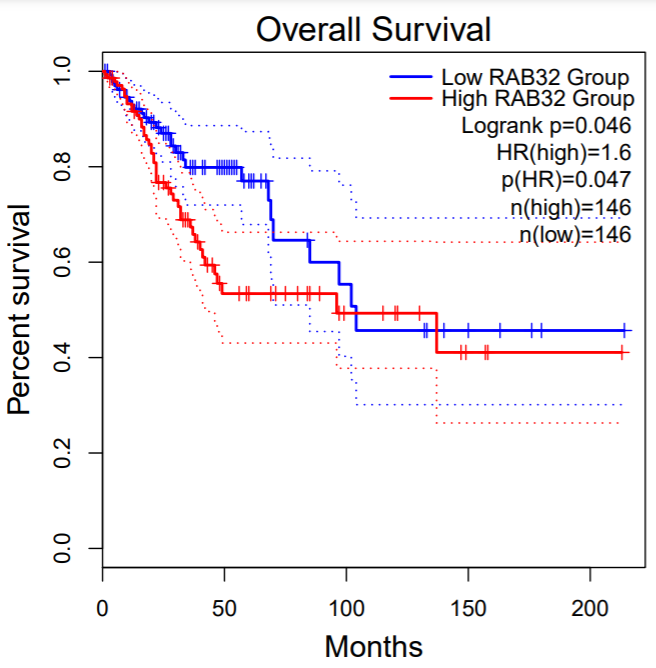

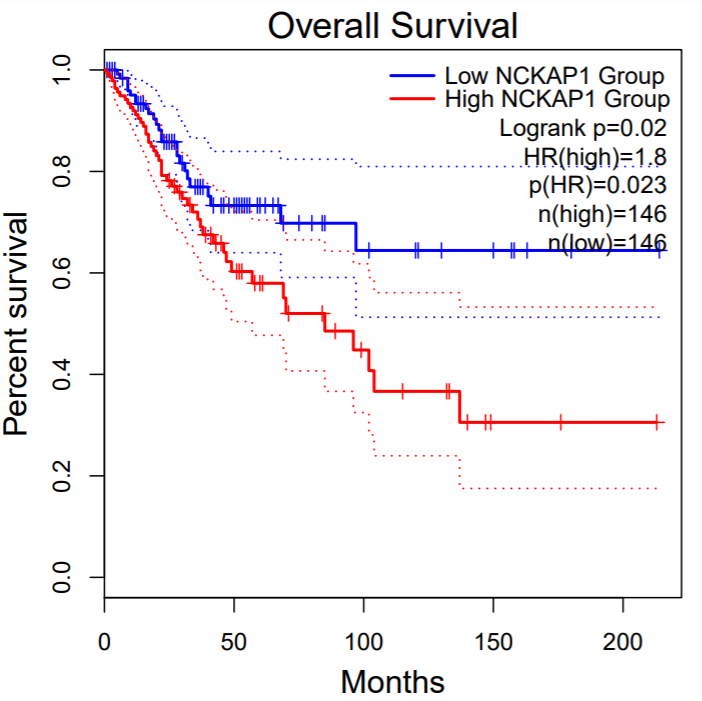


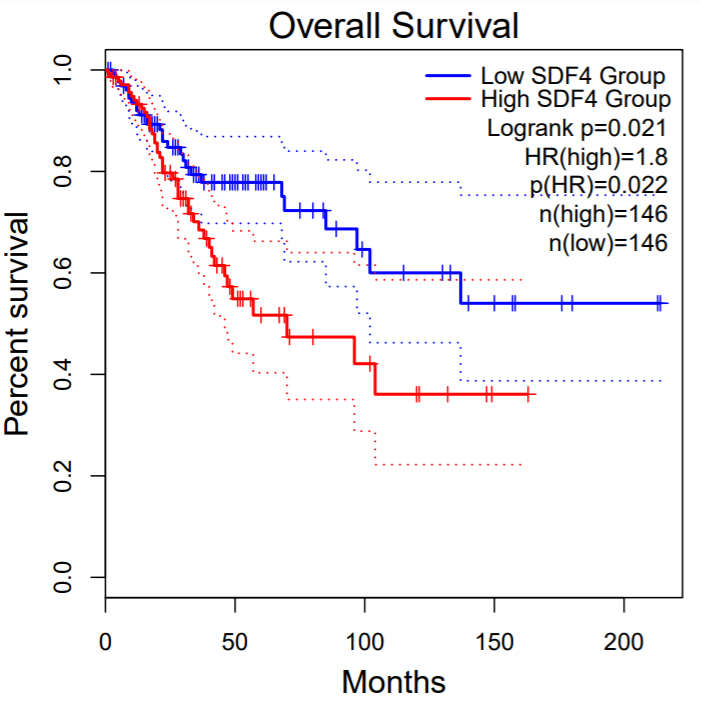

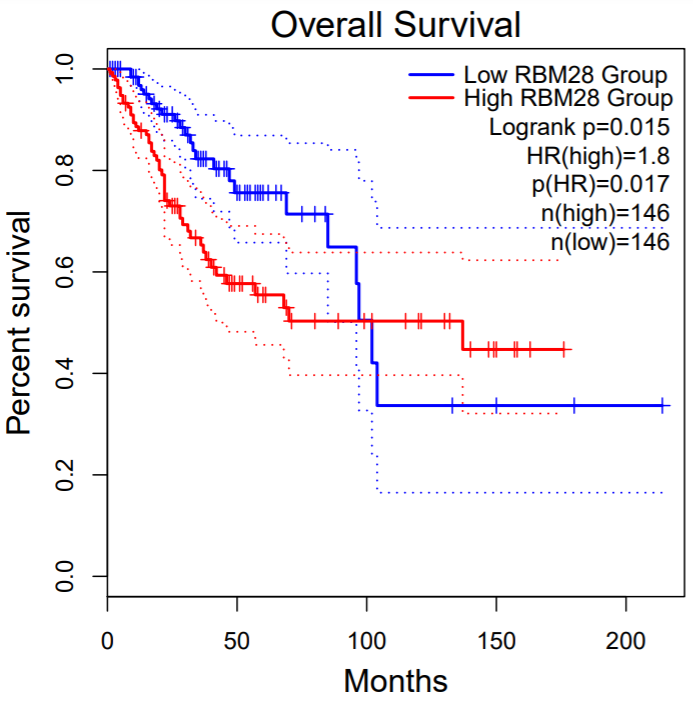


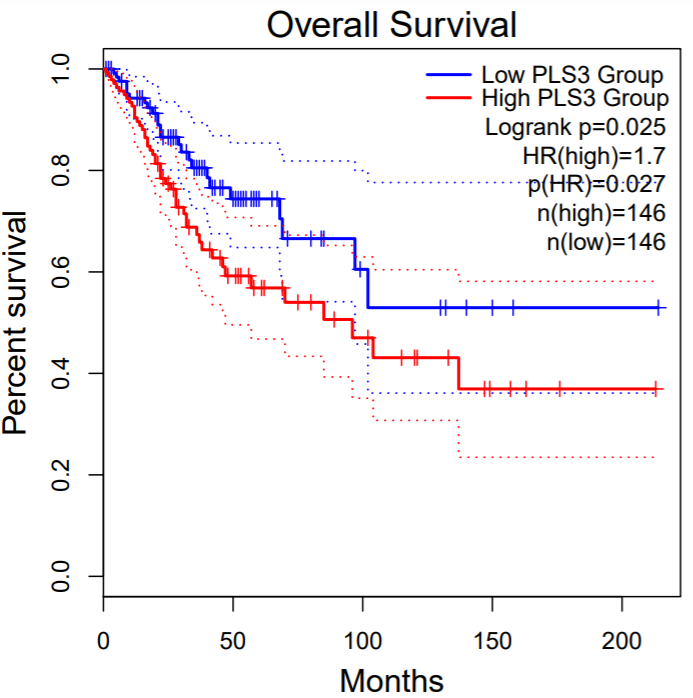

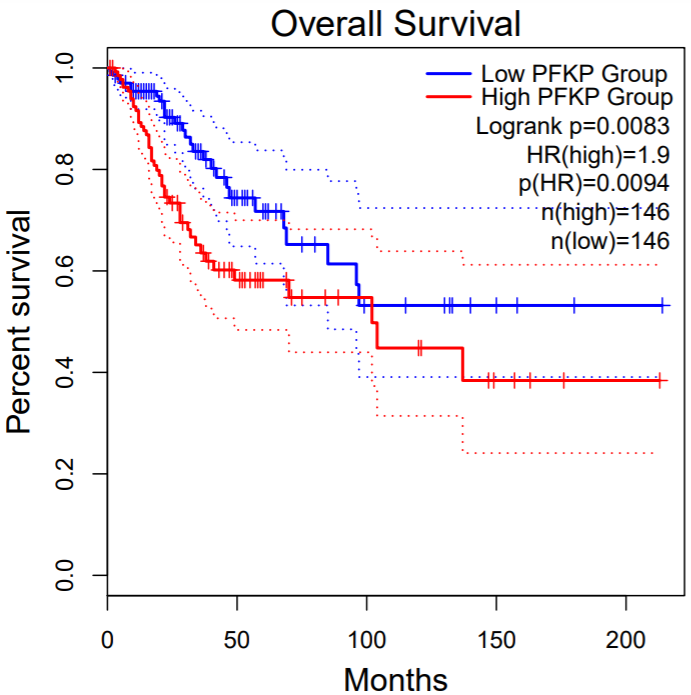


Figure S12 Kaplan-Meier survival curve analysis based on the TCGA CESC database for RAB32, NCKAP1, SDF4, RBM28, PLS3, and PFKP


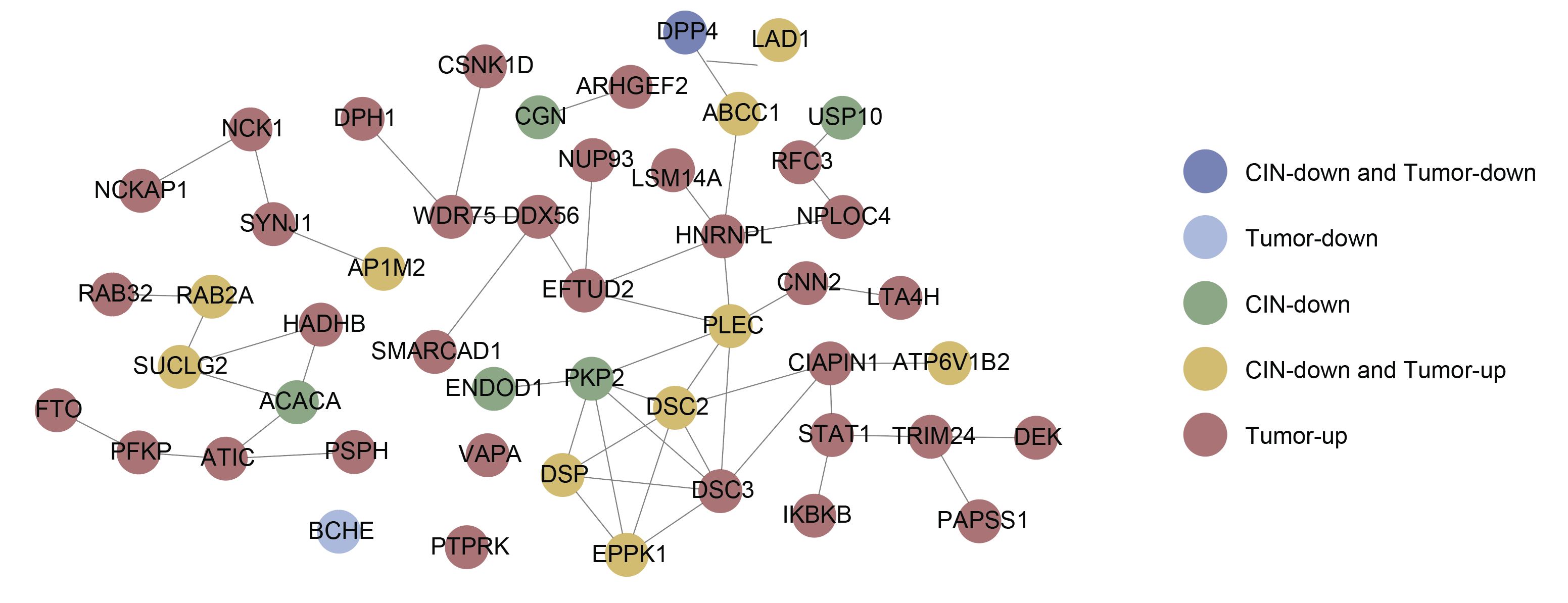


Figure S13 Interaction network of the differentially expressed proteins with HPV integration events obtained with the STRING database.


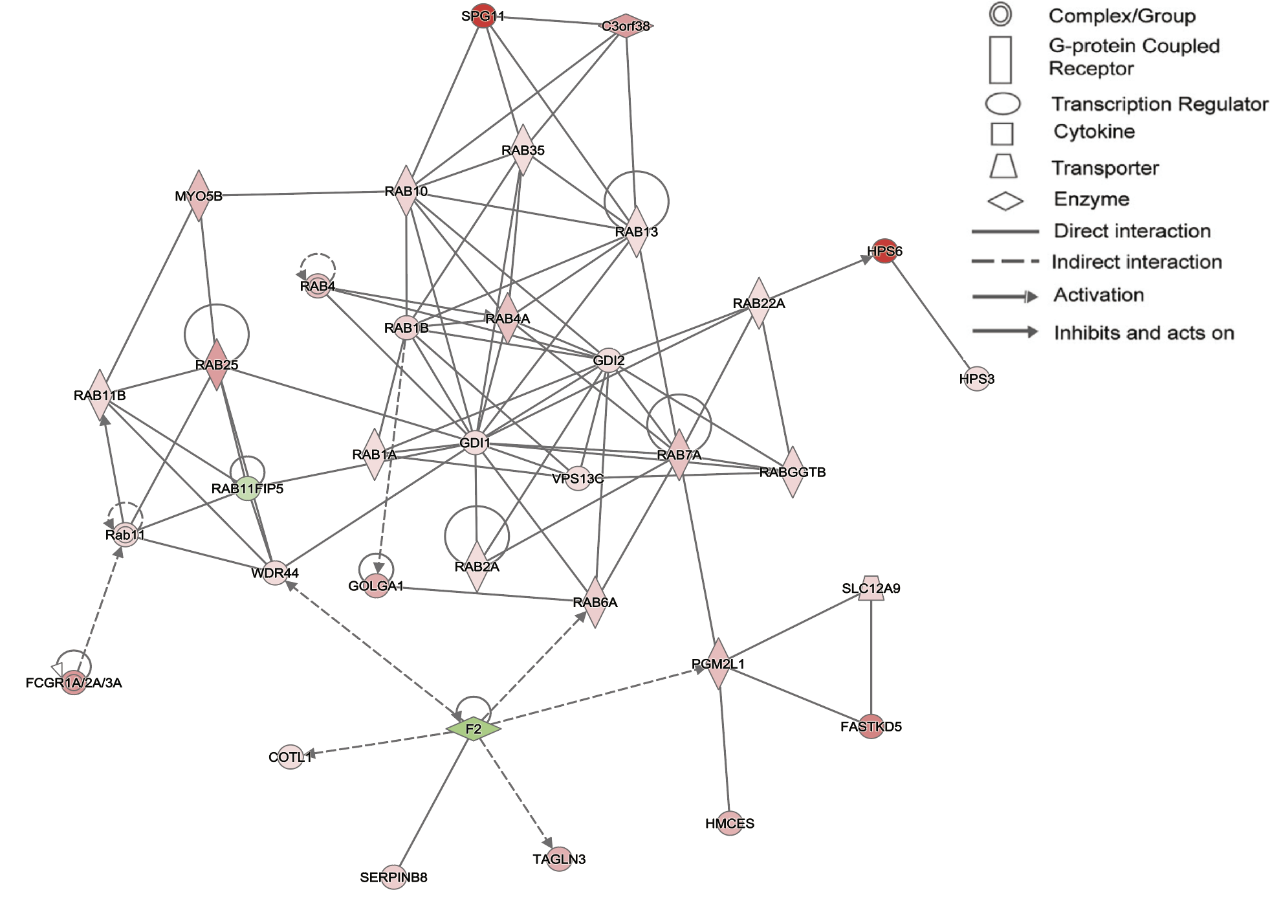
 Figure S14 Graphical representation of the cell-to-cell signaling and interaction network generated with the Ingenuity Pathway Analysis.


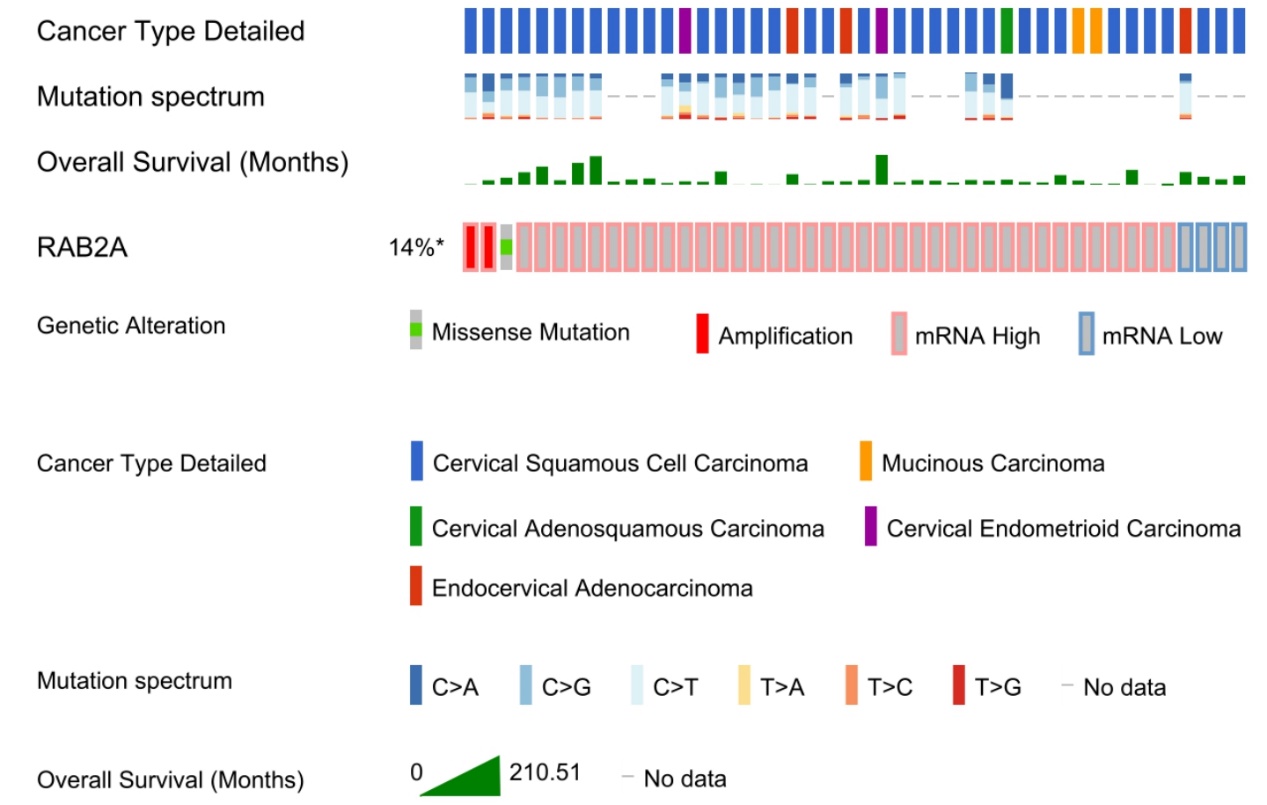


Figure S15 The expression and survival of RAB2A in CESC according to TCGA.

The expression and survival of RAB2A in the cervical squamous cell carcinoma and the endocervical adenocarcinoma (CESC) according to The Cancer Genome Atlas (TCGA). Only the cases with RAB2A mutation or with mRNA-high/low are shown here.


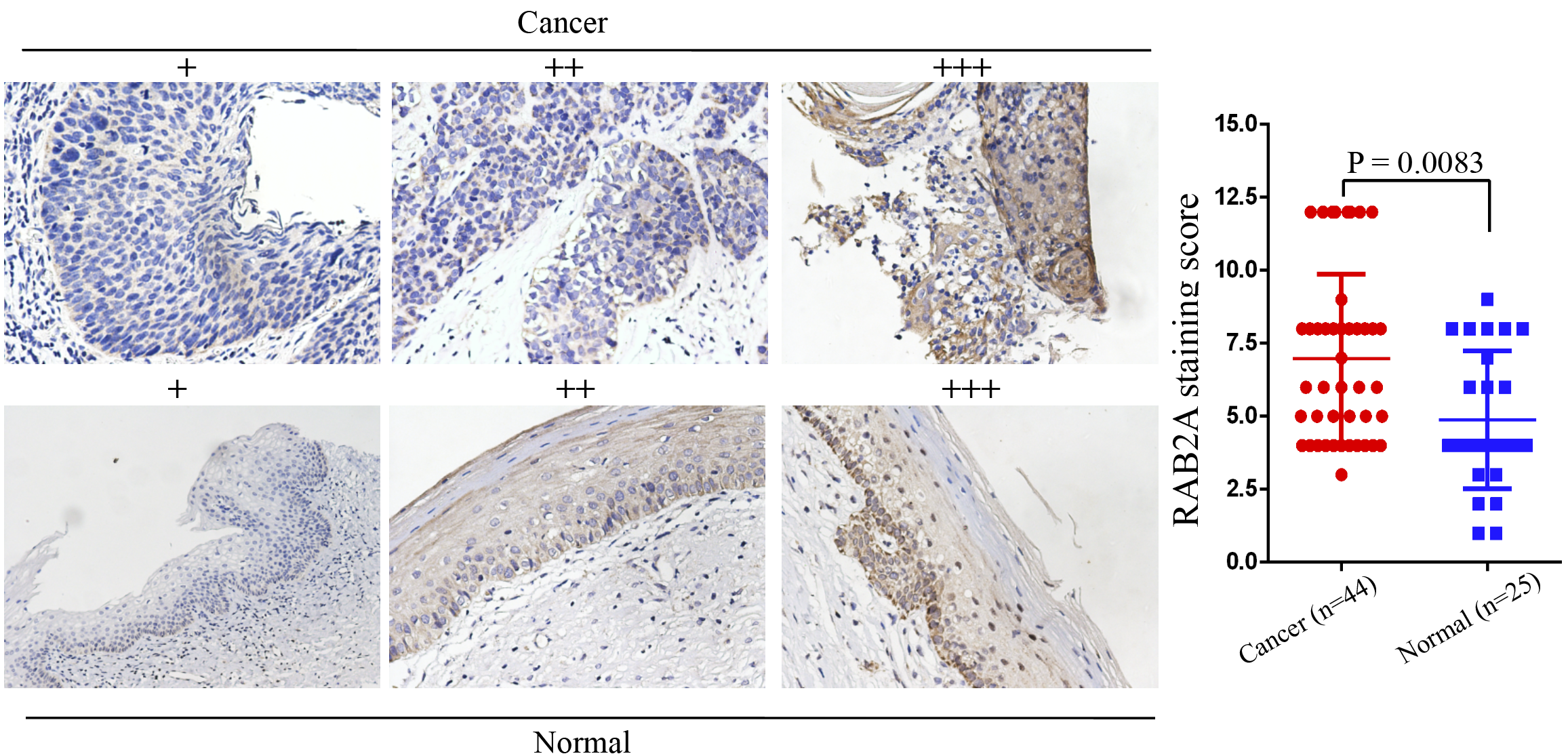


Figure S16 Expression of RAB2A in clinical samples of 25 normal cervix and 44 cancer tissues obtained by immunohistochemistry assay.


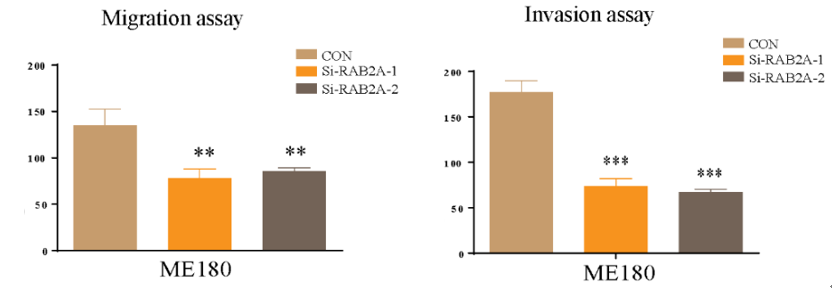


Figure S17 Transwell migration and invasion assays performed in ME180. ** *P* < 0.01 *** *P* < 0.001


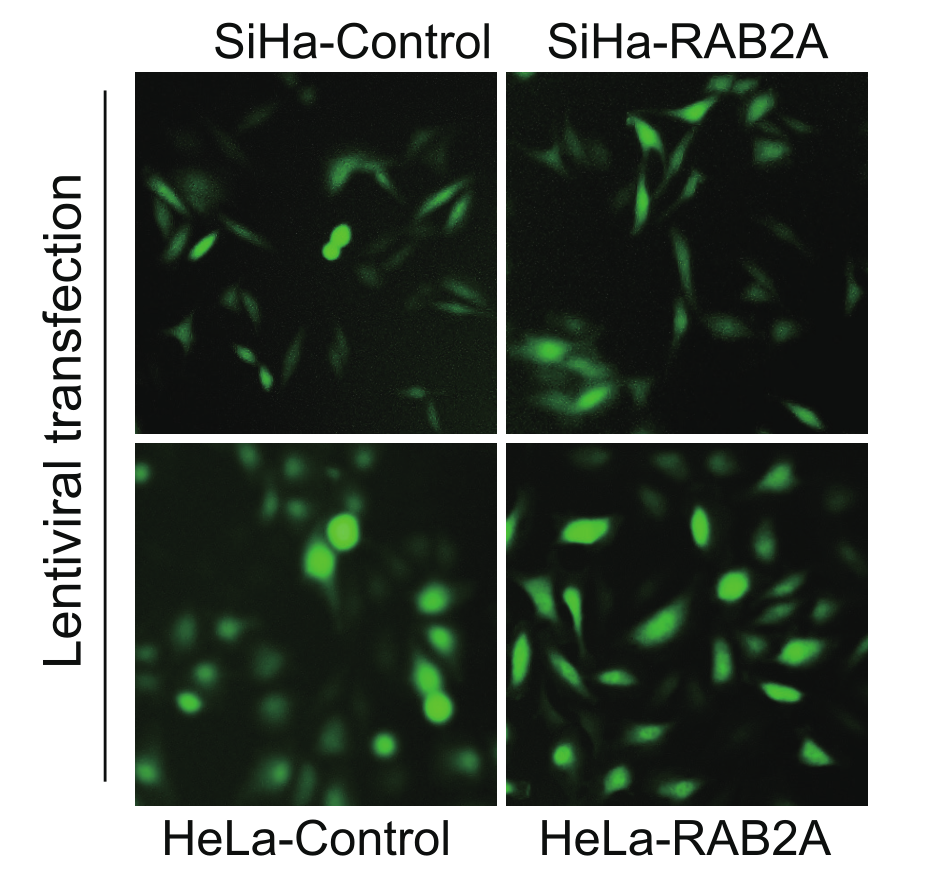


Figure S18 Lentiviral vector expressing green fluorescent protein.


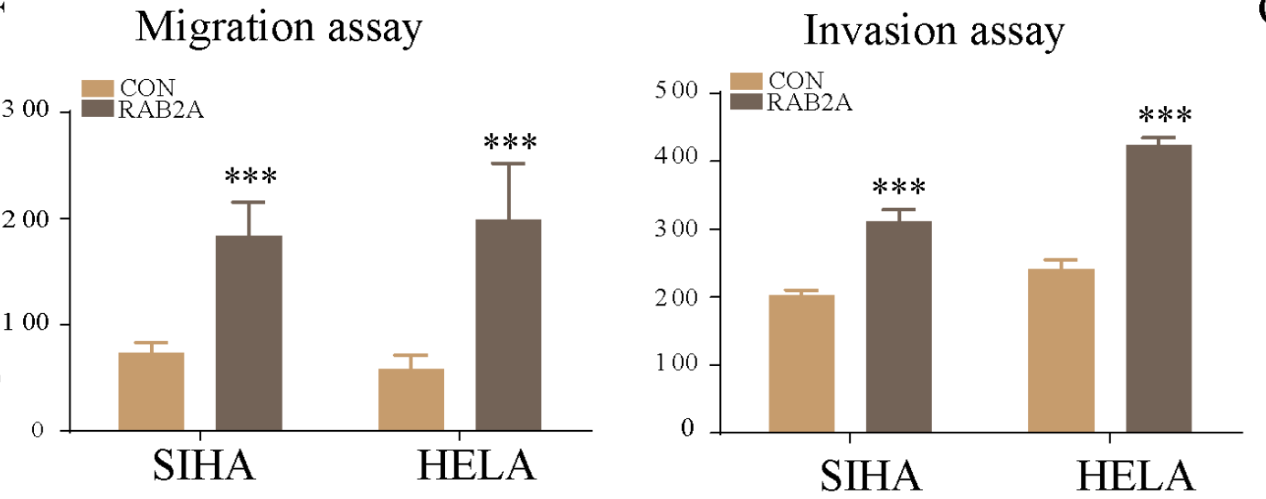


Figure S19 Transwell migration and invasion assays performed in SiHa and HeLa cells. *** *P* < 0.001
